# Isolation of the new polyketide (−)-*R*-talaropinophiloic acid guided by an integrated metabolomics-dereplication approach

**DOI:** 10.1007/s11274-026-05150-2

**Published:** 2026-07-23

**Authors:** Marcus Vinicius Almeida Marques, Andresa Hiromi Sakai, Lucas Haidar Martorano, Viviani N. Takahashi, Juliana Mara Serpeloni, Fernando Martins dos Santos Júnior, Jorge M. David, Eliane O. Silva

**Affiliations:** 1 Department of Organic Chemistry, Institute of Chemistry, Federal University of Bahia, Salvador, Bahia, 40170-115 Brazil; 2 Department of General Biology, Center for Biological Sciences, State University of Londrina, Londrina, 86057-970 Paraná Brazil; 3 Department of Organic Chemistry, Institute of Chemistry, Federal Fluminense University, Niterói, Rio de Janeiro, 24020-141 Brazil; 4 Department of Chemistry, Faculty of Philosophy, Sciences and Letters of Ribeirão Preto, University of São Paulo, Ribeirão Preto, São Paulo, 14040900 Brazil; 5 Department of Organic Chemistry, Institute of Chemistry, Federal University of Bahia (UFBA), Barão de Jeremoabo 147, Salvador, Bahia, 40170-115 Brazil

**Keywords:** Cytotoxicity, Endophytic fungi, *Euphorbia umbellata*, Metabolomics, Polyketides, *Talaromyces pinophilus*

## Abstract

As part of our ongoing bioprospecting program targeting endophytic fungi, the strain *Talaromyces pinophilus* J6 was isolated from *Euphorbia umbellata*. The endophyte metabolic potential was evaluated through a combined metabolomics-dereplication workflow based on an in-house high-resolution mass spectrometry (HRMS) database of *Talaromyces* metabolites. The strategy enabled rapid annotation of seven metabolites (**a**-**g**), allowing rapid characterization of the fungal metabolome and guiding the isolation of potentially new specialized metabolites. Chromatographic fractionation of the crude extract from *T. pinophilus* J6 cultivated on potato dextrose agar supplemented with ammonium sulfate afforded two polyketides: the new (-)-*R*-talaropinophiloic acid (**1**) and the known 3-*O*-methylfunicone (**2**). Their chemical structures were determined by Nuclear Magnetic Resonance (NMR), Circular Dichroism (CD), and HRMS analyses. The cytotoxic activities were assessed against the muscle-invasive bladder cancer cell line T24 by MTT assay, yielding IC₅₀ values of 204.70 µM for **1** and 59.68 µM for **2**, compared with 6.4 µM for the positive control cisplatin. Assays against the non-tumor RPE-1 cell line showed that **2** displayed a CC₅₀ of 16.62 µM, whereas **1** was non-toxic, highlighting **1** as highly selective for cancer cells. Overall, our results expand knowledge of the chemical diversity of the *Talaromyces* genus and underscore its potential as a promising source of new bioactive polyketides.

**Graphical abstract:**

**Figure:**
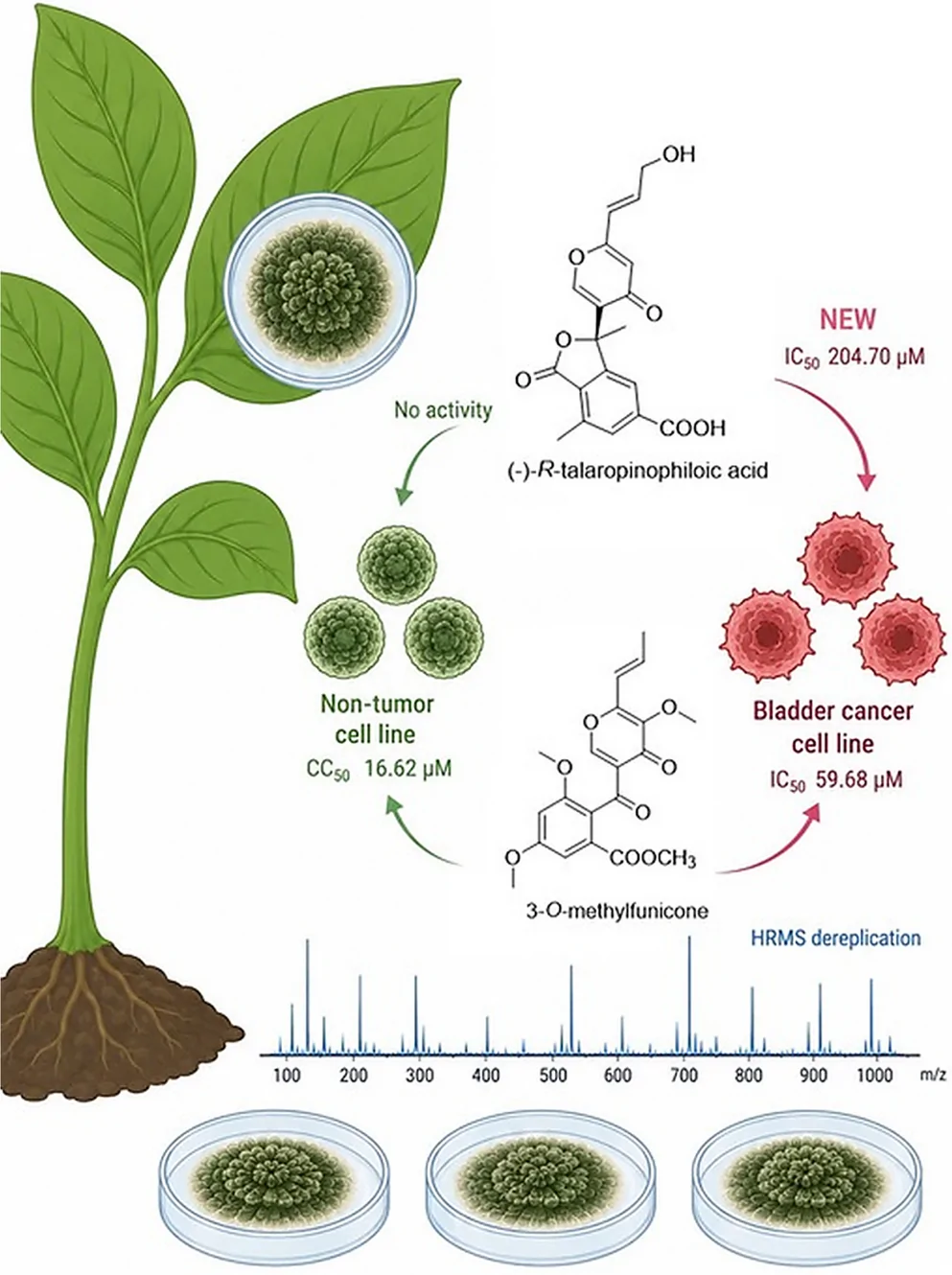
A metabolomics–dereplication approach applied to *Talaromyces pinophilus* J6 from *Euphorbia umbellata* led to the isolation of 3-*O*-methylfunicone and the new (–)-*R*-talaropinophiloic acid with selective cytotoxic activity against T24 cancer cells.

**Supplementary Information:**

The online version contains supplementary material available at 10.1007/s11274-026-05150-2.

## Introduction

Several species of the genus *Euphorbia* (Euphorbiaceae) have demonstrated promising cytotoxic activity with ethnopharmacological relevance (de Oliveira et al. 2013). *Euphorbia umbellata* (formerly *Synadenium umbellatum* or *Synadenium grantii*) is native to Africa and is popularly known in Brazil as “janaúba”. Its latex has been traditionally used in Brazilian folk medicine to treat various types of cancer (da Mota et al. 2012). Furthermore, the antitumoral and antiangiogenic activities of the ethanolic extract obtained from the aerial parts of *E. umbellata* have previously been demonstrated by *in vivo* assays (Nogueira et al. 2008). The development of innovative concepts regarding plant evolution considers the role of the plant-associated microbiome in conferring host capabilities, including growth promotion, improved nutrient uptake, resistance to environmental stresses, and pathogen resistance (Trivedi et al. 2020). Considering the recognized medicinal importance of *Euphorbia* species, their endophytic microbiota represents a promising source of structurally diverse and biologically active metabolites. However, studies focusing on *Euphorbia*-associated microorganisms remain scarce.

The plant endosphere hosts varied endophytic microbial communities that colonize healthy tissues without causing disease for most of their life cycle (Kaul et al. 2012). From establishing symbiotic interactions, endophytic fungi can produce specialized metabolites involved in defense, signaling, and chemical communication (Spiteller 2015), while the host plant provides nutrients and a protected niche (Basit et al. 2021). Endophytic fungi are recognized as prolific sources of structurally diverse and biologically active natural products, including compounds with pharmaceutical and agrochemical relevance, underscoring their significant biotechnological potential as reservoirs of novel chemical scaffolds (Ariantari et al. 2019).

Endophytic fungi inhabit a competitive and stressful natural environment shaped by interactions with coexisting microorganisms, host plant defense mechanisms, and abiotic factors, conditions that stimulate the biosynthesis of specialized metabolites (Chatterjee et al. 2016). These metabolites are typically encoded by biosynthetic gene clusters responsible for multistep biosynthetic pathways (Pfannenstiel and Keller 2019). Under standard laboratory conditions, many of these clusters remain transcriptionally silent due to the absence of specific environmental cues, thereby limiting access to the full endophyte natural product repertoire (Reen et al. 2015). Consequently, conventional cultivation approaches often fail to reproduce the ecological pressures required to trigger bioactive metabolite production, and alternative culture strategies that impose stress to promote the biosynthesis of specialized metabolites are required (Ancheeva et al. 2019).

The OSMAC (One Strain, Many Compounds) strategy (Pan et al. 2019; Zhang et al. 2024b) has been applied to enhance microbial chemical diversity by systematically varying cultivation parameters, including growth medium composition, physicochemical conditions (pH, temperature, and aeration), and the use of chemical or biological elicitors. This approach has proven to be effective in activating otherwise silent biosynthetic pathways, although the rational selection of optimal culture conditions remains a significant challenge. The vast metabolic plasticity of microorganisms across different cultivation conditions renders exhaustive compound identification by traditional approaches impractical. Consequently, modern strategies of natural products analysis, including dereplication and untargeted metabolomics, have become indispensable for the rapid prioritization of novel chemical entities (De Medeiros et al. 2015b; Qin et al. 2022).

In this context, and as part of our ongoing efforts to explore the chemical diversity of endophytic fungi (do Nascimento et al. 2020; Gusmão et al. 2024; Santos et al. 2025), we investigated the metabolic profile of *Talaromyces pinophilus* J6, isolated as an endophyte from *Euphorbia umbellata* leaves. This study reports the isolation of two polyketides, the new (–)-*R*-talaropinophiloic acid (**1**) and the known 3-*O*-methylfunicone (**2**), produced when the fungus was cultured on PDA medium supplemented with ammonium sulfate. Metabolomic profiling by UHPLC/ESI Q-Orbitrap high-resolution mass spectrometry guided the isolation of the aforementioned compounds, whose cytotoxic activities were assayed towards bladder cancer and non-tumoral cell lines. Dereplication against an in-house *Talaromyces* database enabled the putative identification of seven metabolites exclusively detected in the medium containing ammonium sulfate. These findings reinforce the effectiveness of culture-condition manipulation in revealing the hidden biosynthetic potential of endophytic fungi.

## Materials and methods

### Endophytic fungus isolation and identification

The endophytic fungi investigated in the present study were isolated from aerial parts of *Euphorbia umbellata* (Pax) Bruyns (Euphorbiaceae), as previously reported by our research group (Gusmão et al. 2021). Host plant was collected from the Atlantic Forest-Cerrado transition area in Bahia State (S 14°54’46.81’, W 40°48’02.33’) and identified by Dr. Nádia Roque (Institute of Biology, Federal University of Bahia). A voucher specimen was deposited in the Alexandre Leal Costa Herbarium (Federal University of Bahia) under the code ALBC 136,527. The study was registered in the Brazilian System for the Management of Genetic Heritage and Associated Traditional Knowledge (SisGen) under code AE18457.

The strains were preserved in our laboratory and coded as J1, J5, J6, J7, J9, and J13. Prompted by the unique chemical profile displayed by strain J6, it was selected for identification by DNA sequencing followed by phylogenetic analysis. Genomic DNA was extracted from 7-day-old cultures using mechanical disruption with glass microspheres (425–600 μm diameter; Sigma-Aldrich, USA), followed by RNase treatment, phenol: chloroform: isoamyl alcohol (Merck, Germany) extraction, and precipitation with isopropanol, as previously described (Aamir et al. 2015).

PCR amplification was performed using the β-tubulin (benA) gene-specific marker locus with primers Bt2a/Bt2b. PCR reactions were performed in a Thermal Cycler (C1000 Touch, Bio-Rad Laboratories, USA) under conditions previously described (Manganyi et al. 2018). Amplification was confirmed by agarose gel electrophoresis and observed under ultraviolet light before purification. Amplicons were then purified using the GFX PCR DNA and Gel Band Purification Kit (GE Healthcare, USA) and sequenced on an ABI 3500XL Genetic Analyzer (Applied Biosystems, USA). Consensus sequences were manually edited and assembled using BioEdit Sequence Alignment Editor v7.2.6 (Hall 1999), then compared against sequences in the NCBI database with the Basic Local Alignment Search Tool (BLAST; https://blast.ncbi.nlm.nih.gov/). Fungal identification was assigned based on the highest sequence similarity with reference strains deposited in GenBank (https://www.ncbi.nlm.nih.gov/genbank/).

For phylogenetic analysis, sequences were aligned with ClustalX (Thompson 1997) and analyzed in MEGA v.11.0 (Tamura et al. 2021). A phylogenetic tree was reconstructed using the Neighbor-Joining method with the Kimura two-parameter (K2P) model (Kimura 1980), which enabled the calculation of evolutionary distance matrices. Branch support was evaluated with 1,000 bootstrap replicates. The obtained sequence was deposited in GenBank under accession number PQ963936.

The fungal strain has not yet been deposited in a public culture collection. Species identification was inferred from phylogenetic analysis of the benA locus, which showed strong support for its placement within *Talaromyces pinophilus*. Nevertheless, this taxonomic assignment is based on a single genetic marker and should be interpreted accordingly.

### Endophytic fungi cultures and extraction

Endophytic fungal strains J1, J5, J6, J7, J9, and J13 were cultivated in Petri dishes containing 20 mL of three distinct culture media: (i) Potato Dextrose Agar (PDA, Kasvi, Brazil), (ii) PDA supplemented with 45% (w/w) sodium tartrate dihydrate (Merck, Germany) (PDA_ST), or (III) PDA supplemented with 45% (w/w) ammonium sulfate (Merck, Germany) (PDA_AS). All cultures were prepared in triplicate and incubated simultaneously in a BOD (Biochemical oxygen demand) chamber at 28 °C for 10 days. Control (blank) extractions were performed under identical conditions using PDA, PDA_ST, or PDA_AS without fungal inoculation.

After incubation, crude extracts were obtained by adding 40 mL of ethyl acetate (EtOAc, Synth, Brazil) directly to each culture plate (including both the medium and the mycelial mass). The mixtures were sonicated for 20 min, filtered, and the organic soluble fraction was concentrated to dryness under reduced pressure (Ika, Germany) at 40 °C.

### UHPLC-HRMS fungal extracts analysis

All crude extracts from endophyte cultures were analyzed by ultrahigh-performance liquid chromatography coupled with high-resolution mass spectrometry (UHPLC-HRMS). MS1 and MS2 data were acquired using data-dependent acquisition (DDA) in both positive and negative ionization modes, with the 10 most intense features selected. The dereplication workflow was based on accurate mass (MS1) information, supported by feature-specific MS2 data.

The UHPLC-HRMS equipment (Thermo Fisher Scientific, USA) consisted of an electrospray ionization (ESI) source and an Orbitrap analyzer. The flow rate was 400 µL/min, and the gradient elution system was 5 to 100% methanol (HPLC grade Tedia, USA) in water for over 30 min. The C18 column (ACE 150 mm × 4.6 mm ×3 μm, Nova Analítica, Brazil) temperature was set to 30 °C. The following parameters were used: scanning range of 120–1200 *m*/*z* for full MS, ESI MS resolution of 70.000 with lock mass, microbeam of 1, and maximum injection time of 250 ms. The parameters of the ESI ionization source were set with a 30 L/min gas flow rate; positive voltage spray mode of 3.6 kV; negative voltage spray mode of 3.2 kV; and Slens level of 55. Nitrogen gas was used as a nebulizer in the collision cell. The mass spectra were processed using Xcalibur software (Thermo Fisher Scientific, USA).

One microliter of each sample (1 mg/mL) was randomly analyzed. The results from the sample replicates (*n* = 3), and a blank analyzed at the beginning, middle, and end of the sequence, were evaluated to ensure analytical consistency and instrument performance.

### Dereplication and Metabolomics

The raw mass data were converted to .mzXml format using MSconvert software (ProteoWizard Software Foundation, USA). In the sequence, the chromatograms of the different extracts, with mass detection in positive ionization mode, were processed using MZmine 4.5.0 (MZmine Development Team). All base peak chromatograms (BPC) data were inspected to verify chromatographic consistency and reproducibility across replicates before proceeding to feature extraction. The centroid data were resolved, isotopes were removed, similar peaks across chromatograms were aligned, blank features were subtracted, and gaps were filled. MZmine parameters were set as follows. Mass detection: noises 2.0E7 and 4.0E6 for MS1 and MS2 levels, respectively. The ADAP algorithm was employed for chromatogram builder with 5 minimum consecutive scans, 6.0E7 minimum intensity for consecutive scans, 4.0E7 minimum absolute height, 0.002 *m*/*z* or 10.0 ppm tolerance (scan-to-scan). Data resolution using local minimum feature resolver: 0.01 or 10.0 ppm precursor tolerance, 0.06 min retention time filter, 0.85 chromatographic threshold, 0.05 minimum search range RT/Mobility (absolute), 5E5 minimum absolute height, 2.0 minimum ratio of peak top/edge, 5 minimum scans. ^13^C Isotope filter: 0.01 *m*/*z* or 3.0 ppm tolerance (intra-sample), 0.02 min retention time tolerance, 2 maximum charges, most intense for representative isotope. Features alignments using the Join aligner algorithm: 0.01 *m*/*z* or 5.0 ppm tolerance (sample-to-sample), 3.0 weight for *m*/*z*, 0.06 min retention time tolerance, 1.0 weight for RT. Gap filling: 0.02 *m*/*z* or 5 ppm tolerance, 20% intensity tolerance, 0.06 min retention time tolerance, 3 minimum scans. Blank subtraction was performed directly within the MZmine data-processing workflow, where features detected in the blank were removed from the dataset prior to downstream analyses.

After feature processing and generation, a dereplication workflow was applied using a curated in-house database comprising 773 metabolites previously reported for the genus *Talaromyces*. This database contains molecular formulae and accurate masses compiled from research articles and reviews. Dereplication was performed by integrating the in-house library directly into the MZmine platform. The scores reported in Table S1 correspond to the similarity metric generated by the MZmine platform and are based on the agreement between the experimentally measured accurate masses of the detected features and those of compounds present in the in-house database. Higher scores indicate a closer match between the observed and expected masses, thereby increasing confidence in the putative annotation. A mass accuracy tolerance of 5 ppm (error) was applied during the annotation process. MS2 spectra corresponding to features with a matching score above 0.5 and containing [M + H]⁺ or [M + Na]⁺ adducts were then carefully inspected (first column of Table S1). Their fragmentation patterns were proposed based on diagnostic fragment ions and neutral losses (MS2 spectra). To comply with established metabolomics standards, putative annotations of metabolites **a**-**g** were assigned at confidence level 2 according to the Metabolomics Standards Initiative (Creek et al. 2014). This approach enabled rapid annotation of known compounds and facilitated the prioritization of potentially novel metabolites during metabolic profiling.

To visualize sample grouping, an unsupervised multivariate data analysis was performed using MetaboAnalyst 6.0 software (https://www.metaboanalyst.ca). The multivariate statistical analysis was used to explore the dataset, enabling discrimination among samples and among metabolite attributes. Three biological replicates per experimental condition were analyzed. We performed statistical analyses using the metabolite matrix via Principal Component Analysis (PCA). The data were normalized by median, log-transformed, and scaled by mean centering. No pooled quality control (QC) samples or internal standards were included in the UHPLC-HRMS analytical sequence because the objective of this study was comparative metabolite prioritization rather than quantitative metabolomics. Consequently, the multivariate analysis was used as an exploratory tool, and the lack of QC samples and internal standards constitutes a methodological limitation.

All raw data used are available in the MassIVE repository (MSV000102064).

### Isolation and structural identification of the specialized metabolites

Supported by the chemical uniqueness presented by the J6 strain (shown by metabolomics analysis), such an endophytic fungus was cultivated in 100 Petri dishes (90 mm) containing PDA medium supplemented with ammonium sulfate (45% w/w) and incubated in a BOD chamber for 10 days at 28 °C. Specialized metabolites were extracted with 2000 mL of EtOAc, followed by 20 min of sonication and filtration. The resulting extract was dried over anhydrous sodium sulfate and concentrated under reduced pressure at 40 °C, yielding 1300 mg of scale-up crude extract. The purification process was carried out on a silica gel 60 A (Sigma-Aldrich, USA) chromatographic column (40 × 1.5 cm). The mobile phase consisted of hexane (Synth, Brazil), EtOAc (Synth, Brazil), and methanol (MeOH, Synth, Brazil) in a gradient elution. A total of 10 fractions of 300 mL were collected. The ninth fraction (EtOAc: MeOH 1:1) was subjected to further purification by solid-phase extraction (SPE) using a Visiprep-SPE (Supelco, USA) system with a reverse-phase column. The sample was added to C_18_ cartridge, which was eluted with increasing portions of MeOH in water. Polyketides **1** and **2** were achieved as pure compounds (17.5 mg and 22.0 mg, respectively) from fractions eluted with 100% MeOH and 70% MeOH in water, respectively.

One- and two-dimensional NMR spectra were recorded at 500 MHz and 125 MHz for ^1^H and ^13^C, respectively, using a DRX 500 spectrometer (Bruker, USA). Chemical shifts (*δ*) were referenced to the residual deuterated methanol (CD_3_OD, Cambridge Isotope Laboratories, USA) peaks at *δ*_H_ 3.31 for ^1^H and *δ*_C_ 49.00 for ^13^C.

Circular dichroism (ECD) was employed for the absolute stereochemistry determination of compound **1**. The optical rotation was measured in a YK-P100 digital automatic polarimeter (Yuke Jiahang, China) using the following parameters: acetonitrile (ACN, Merck, Germany) solution (1.0 mg/mL), cell path length (50 mm), and 20 °C, with 3 accumulations. The ECD spectrum was recorded with a Jasco J-1100 spectrometer (Jasco, Japan) in the 190–400 nm region using the following parameters: bandwidth 1 nm; response 1s; scanning speed 100 nm/min; 3 accumulations; 0.1 cm cell path length. For the simulation of ECD spectra, randomized conformational searches were performed for the three-dimensional molecular structures of compound **1** using the Monte Carlo algorithm with a Merck molecular force field (MMFF) in Spartan’14 software (wavefunction Inc, USA) (Origin(Pro), 2023). A total of 73 conformers within a relatively free energy window of 10 kcal/mol were selected for geometric optimization using the B3LYP/6‑31G(d) level of theory. The polarizable continuous model with integral equation formalism (IEF-PCM) to implicitly simulate ACN as the solvent. Vibrational frequency calculations were performed at the same level of theory to confirm that the stationary points correspond to minimum on the potential energy surface. Subsequently, 41 conformers with relative energies up to 2,5 kcal/mol, which correspond to more than 98% of the total Boltzmann distribution, were selected for ECD simulations. These ECD calculations were conducted at the TD‑DFT level of theory: CAM-B3LYP/TZVP, also employing the IEF-PCM to simulate ACN as the solvent implicitly. The final ECD spectra were generated based on Boltzmann statistics for the selected conformers and plotted using Origin 8 software (OriginLab Corporation, USA). All quantum-mechanical calculations were performed using the Gaussian 09 software package (Gaussian Inc, USA) (Frisch et al. 2009).

(*R*,*E*)-1-(6-(3-Hydroxyprop-1-en-1-yl)-4-oxo-4*H*-pyran-3-yl)-1,7-dimethyl-3-oxo-1,3 dihydroisobenzofuran-5-carboxylic acid or (-)-*R*-talaropinophiloic acid (**1**): white powder; ^1^H-NMR (500 MHz, CD_3_OD) and ^13^C-NMR (125 MHz, CD_3_OD) data, see Table 2; [α]^25^_D_ = − 6.0° (*c* 0.1, acetonitrile). HRMS *m*/*z* 357.0983 ([M + H]^+^; (C_19_H_16_O_7_)H^+^; calc. 357.0969; error 3.9 ppm).

(*E*)-3-methoxy-2-propenyl-5-(2′-carbomethoxy-4′-6′-dimethoxybenzoyl)-4-pyrone or 3-*O*-methylfunicone (**2**): white powder; ^1^H-NMR (500 MHz, CD_3_OD): *δ* 8.48 (1H, *s*, H-9); 7.07 (1H, *d*, *J* = 2.0 Hz, H-6); 6.81 (1H, *d*, *J* = 2.0 Hz, H-4); 6.74 (1H, *d*, *J* = 6.7 Hz, H-15); 6.61 (1H, *dd*, *J* = 15.8 and 1.7 Hz, H-14), 3.88 (3 H, *s*, 5-OCH_3_), 3.78 (3 H, *s*, 7-COOCH_3_), 3.76 (3 H, *s*, 12-OCH_3_), 3.75 (3 H, *s*, 3-OCH_3_), 1.97 (3 H, *dd*, *J* = 6.7 and 1.7 Hz, H-16); ^13^C-NMR (125 MHz, CD_3_OD): *δ* 192.8 (C-1), 174.3 (C-11), 168.0 (7-COO), 163.1 (C-5), 161.5 (C-9), 159.5 (C-3), 156.8 (C-13), 145.2 (C-12), 137.4 (C-15), 132.0 (C-7), 128.0 (C-10), 126.0 (C-2), 119.3 (C-14), 107.3 (C-6), 103.6 (C-4), 61.1 (12-OCH_3_), 56.7 (3-OCH_3_), 56.3 (5-OCH_3_), 52.9 (7-COOCH_3_), 19.0 (C-16). HRMS *m*/*z* 389.1244 ([M + H]^+^; (C_20_H_20_O_8_)H^+^; calc. 389.1231; error 3.3 ppm).

### Cytotoxic assays

The experimental model was the bladder tumor cell line T24 (ATCC^®^ HTB-22™). For comparison, a normal retinal pigment epithelial cell line immortalized by hTERT (RPE-1) (ATCC^®^ CRL-4000) was also assayed. T24 cells were cultured in Roswell Park Memorial Institute 1640 medium (RPMI 1640, 11875093, Gibco, USA). The cells and RPE-1 cells were cultivated in Dulbecco’s Modified Eagle Medium/Nutrient Mixture F-12 (DMEM/F-12, 12500062, Gibco, USA). Both cell cultures were supplemented with 10% sterile triple-filtered fetal bovine serum (FBS, Gibco, USA) (10-bio500, Nova Biotecnologia, Brazil) and 1% antibiotic–antimycotic solution (100X; 15240062). The cells were grown in 25 cm^2^ cell culture bottles at 37 °C in an atmosphere containing 5% CO_2_ and 95% relative humidity. After reaching 80–90% confluence, the cells were sub-cultured. All experiments were performed using aliquots from the 3rd to the 8th cell passage. The cells were treated with different concentrations of compounds **1** and **2** (10, 50, 100, 200, 300, 400, and 500 µM), solubilized in dimethyl sulfoxide (DMSO 0.25%, final concentration in culture). Cisplatin (232120, Merck, Germany) was used as a positive control at a concentration of 6.4 µM, as previously standardized.

Evaluation of cell viability through the MTT, 3-(4,5-dimethylthiazol-2-yl)-2,5-diphenyltetrazolium bromide, reduction assay (M6494; Thermo Fisher Scientific, USA) was performed according to a previous protocol (Mosmann 1983) after 24 h of treatments. After treatments, a MTT working solution (0.015 mg/mL phosphate-buffered saline, PBS) was added to each well at 20% of the final volume (40 µL). The plates were incubated for 4 h at 37 °C, and absorbance was measured in a microplate reader (Biotek Eon, USA) at 540 nm. Cell viability was calculated by normalizing the absorbance of each treatment to the solvent control (SV), which was set at 100%.

All the experiments described were performed in three biological replicates (*n* = 3). The results were analyzed using analysis of variance (ANOVA), followed by Dunnett’s test, with *p* ≤ 0.05 considered significant. All statistical analyses were performed using the GraphPad Prism 7.0 software. Selectivity index (SI) was calculated as CC_50_ RPE-1 / IC_50_ T24.

## Results

### Untargeted metabolomics to access metabolic differences among endophytic fungi

Six endophyte strains isolated from the aerial parts of *Euphorbia umbellata*, coded as J1, J5, J6, J7, J9, and J13, were grown in Petri dishes containing: (i) Potato Dextrose Agar (PDA), (ii) PDA supplemented with 45% (w/w) sodium tartrate dihydrate (PDA_ST), or (iii) PDA supplemented with 45% (w/w) ammonium sulfate (PDA_AS). All ethyl acetate extracts from endophytic cultures were analyzed by UHPLC/ESI Q-Orbitrap, and their base peak chromatograms (BPCs) are shown in Figure S1 (Supplementary Material). The positive ionization mode yielded higher signal intensity and more detected features. Therefore, it was selected for untargeted metabolomic analyses.

**Fig. 1:**
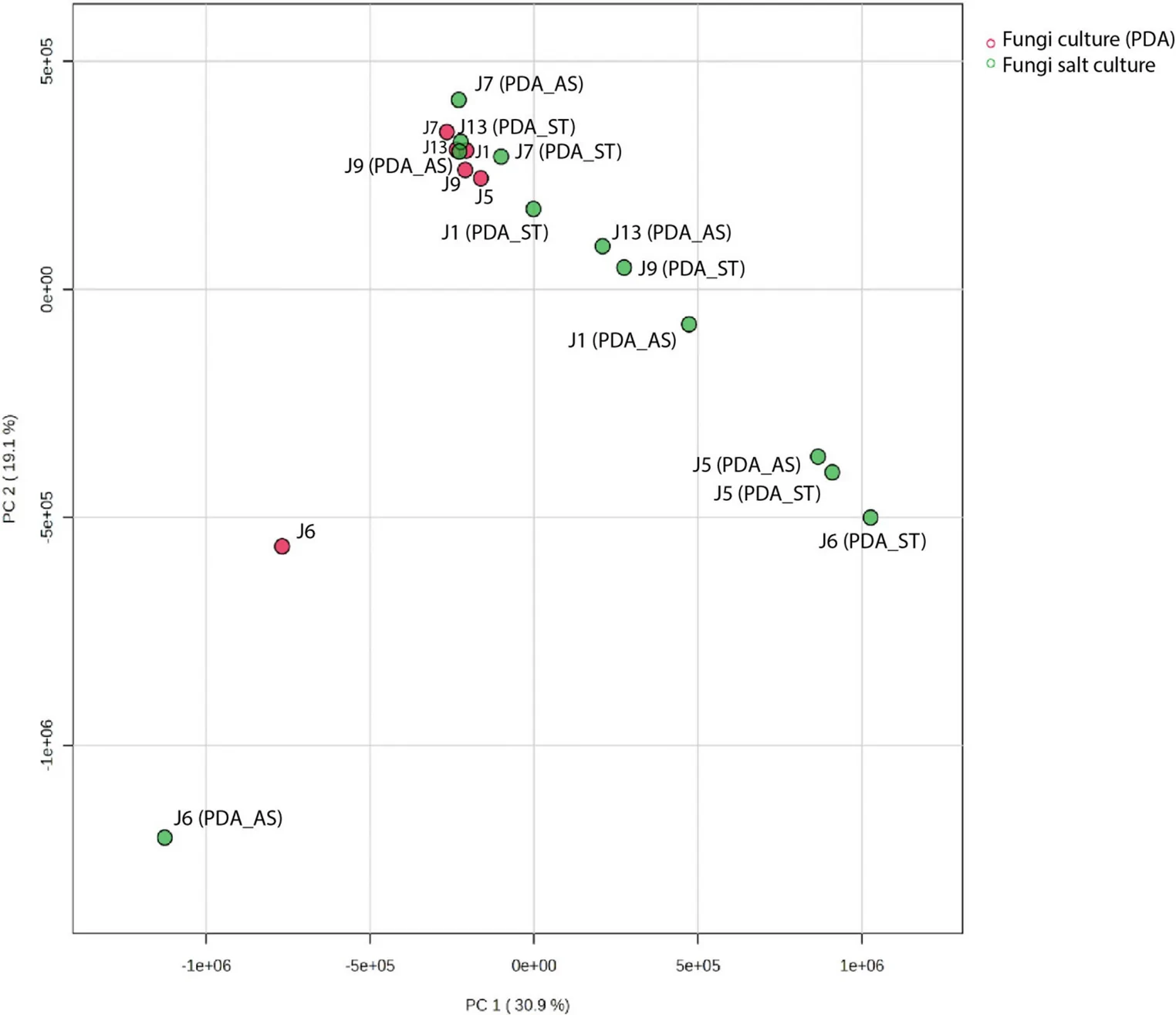
PCA scores plot (PC1 vs. PC2 from five components) for the multivariate association between chemical profiles of crude extracts of six endophytic strains (J1, J5, J6, J7, J9, and J13) isolated from *E. umbellata* and submitted to PDA or PDA salt cultures. Strains and culture conditions, designated by color, were clustered based on natural products detected by UHPLC-HRMS

Unsupervised multivariate statistical analysis was performed using Principal Component Analysis (PCA). According to the PCA models, five principal components were gained by comparing the metabolic profiles of J1, J5, J6, J7, J9, and J13 strains grown on PDA, PDA_ST, and PDA_AS. The PCA scoring plots showed the clustering pattern outcomes, and the first two principal components explained 50% of the overall variance. The first principal component (PC 1) had 30.9% of the total variation, and 19.1% of the total variance was contributed by the second principal component (PC 2) (Fig. 1).

Among all analyzed samples, the crude extracts of strain J6 grown on PDA and PDA_AS were clearly separated from those of the other fungi, clustering in the lower-left quadrant of the PCA score plot. Notably, the PDA_AS culture of J6 showed the greatest separation, indicating a unique metabolite profile compared with the other samples.

### Molecular identification of the endophytic fungus J6 and chemical dereplication of its extract

The selected J6 strain exhibited macroscopic characteristics consistent with those previously reported for *Talaromyces* spp. Despite the osmotic stress imposed by the saline medium, J6 demonstrated satisfactory growth under both conditions (PDA and PDA_AS), indicating its ability to tolerate elevated salt concentrations. Representative 7-day cultures grown in the presence and absence of salt are shown in Fig. S2 (Supplementary Material).

**Fig. 2:**
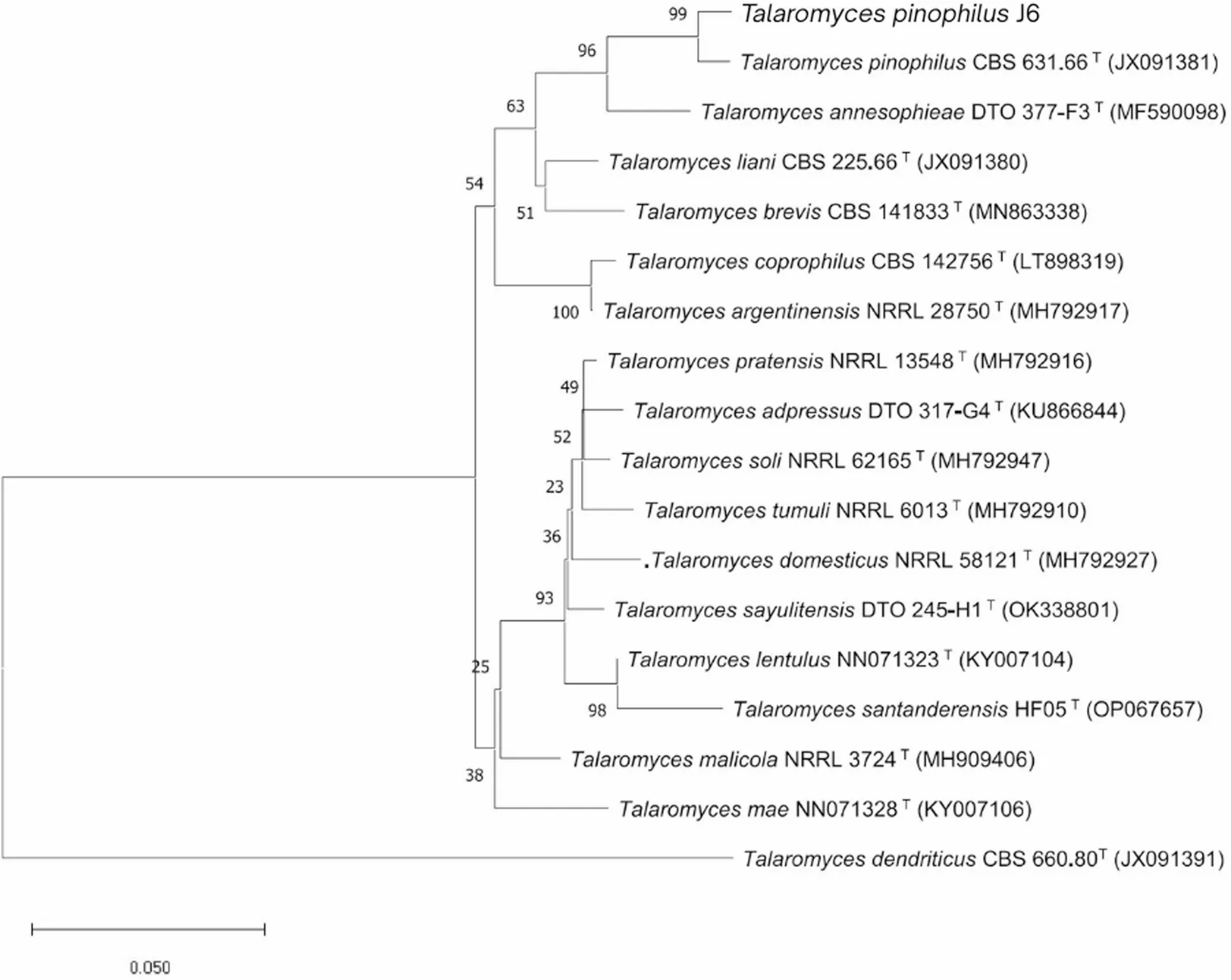
Phylogenetic position based on benA sequence of *Talaromyces pinophilus* J6. The phylogenetic tree was constructed by the Neighbor-Joining approach with MEGA 6. Bootstrap analysis was performed using 1,000 replications, and it is indicated at the nodes. The scale bars represent 0.050 substitutions per site

In the sequence, strain J6 was identified using molecular approaches. Phylogenetic analysis based on the benA gene sequence positioned J6 among closely related taxa, as illustrated in the phylogenetic tree shown in Fig. 2. The results indicated that J6 belongs to the genus *Talaromyces* and is closely related to *T. pinophilus*, forming a well-supported clade with *T. pinophilus* CBS 631.66 (JX091381) with a bootstrap value of 99%.

**Fig. 3:**
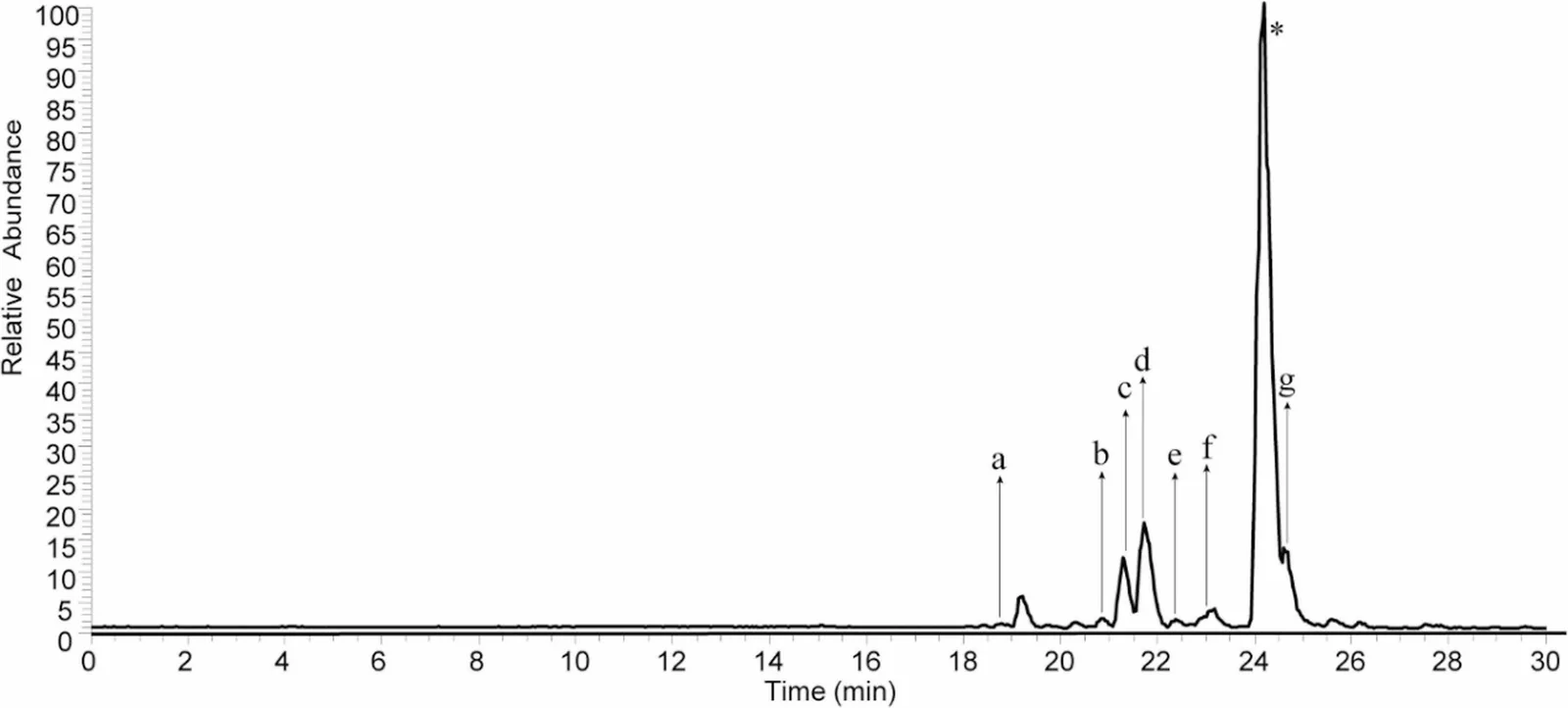
Base peak chromatogram (100–1000 Da) of the *Talaromyces pinophilus* J6 extract obtained from cultures grown in PDA_AS medium. Compounds annotated by HRMS-based dereplication are indicated: 3,4-dihydroxyphenylacetic acid methyl ester (**a**), 3-(hydroxymethyl)-6,8-dimethoxy-2*H*-chromen-2-one (**b**), talaromycolide A (**c**), actofunicone (**d**), amestolkin (**e**), funicone (**f**), rubralide C (**g**). Compounds **a**-**g** were exclusively detected in the PDA_AS medium (see Table S1). The two isolated compounds, (–)-*R*-talaropinophiloic acid (**1**) and 3-*O*-methylfunicone (**2**), co-eluted and are indicated (*). Data were acquired using electrospray ionization in positive ion mode

Next, a dereplication workflow was applied to comprehensive putative chemical characterization of crude extracts from *T. pinophilus* J6 (PDA, PDA_ST, and PDA_AS). A curated in-house database comprising 773 metabolites previously reported for the *Talaromyces* genus (Pereira et al. 2026) was used in the dereplication by integrating the in-house library directly into the MZmine platform. Our dereplication strategy led to the annotation of 94 specialized metabolites, whose biosynthesis occurred exclusively when *T. pinophilus* J6 was cultured in the PDA_AS medium (Feature status as ‘detected’ only in PDA_AS, Table S1 Supplementary Material). Among these 94 compounds, a critical investigation was conducted using the observed accurate monoisotopic masses, fragment ions, and adduct patterns. During the annotation process, features with a mass error tolerance of ≤ 5 ppm, an MZmine matching score greater than 0.5 against our in-house library, and common [M + H]⁺ or [M + Na]⁺ adducts were selected. As a result, seven natural products (**a**-**g**) were prioritized for tentative annotation. Their annotation was initially supported by MS1 data and subsequently strengthened through MS2 spectral analysis and the proposal of fragmentation pathways. Their proposed structures were supported by the interpretation of diagnostic fragment ions and neutral losses observed in the MS2 spectra. An overview of these seven putatively annotated compounds (**a**–**g**), exclusively detected in *T. pinophilus* J6 cultivated in PDA_AS medium, is shown in Fig. 3.

**Fig. 4:**
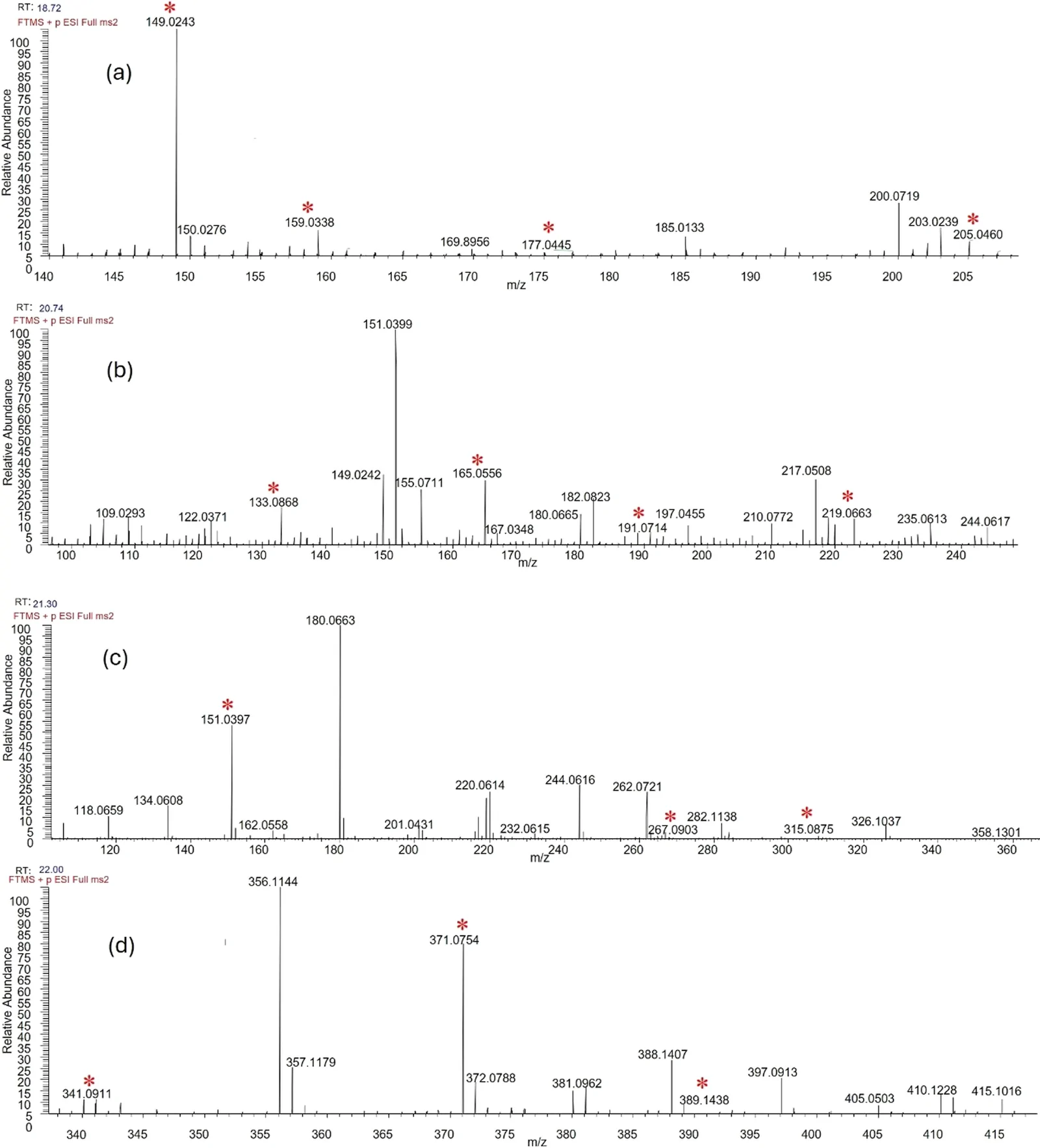
MS2 spectra acquired in positive ion mode of 3,4-dihydroxyphenylacetic acid methyl ester (**a**), 3-(hydroxymethyl)-6,8-dimethoxy-2*H*-chromen-2-one (**b**), talaromycolide A (**c**), and actofunicone (**d**). The diagnostic fragment ions proposed in the fragmentation scheme (Fig. 5) are highlighted in the spectrum with red asterisks

**Fig. 5:**
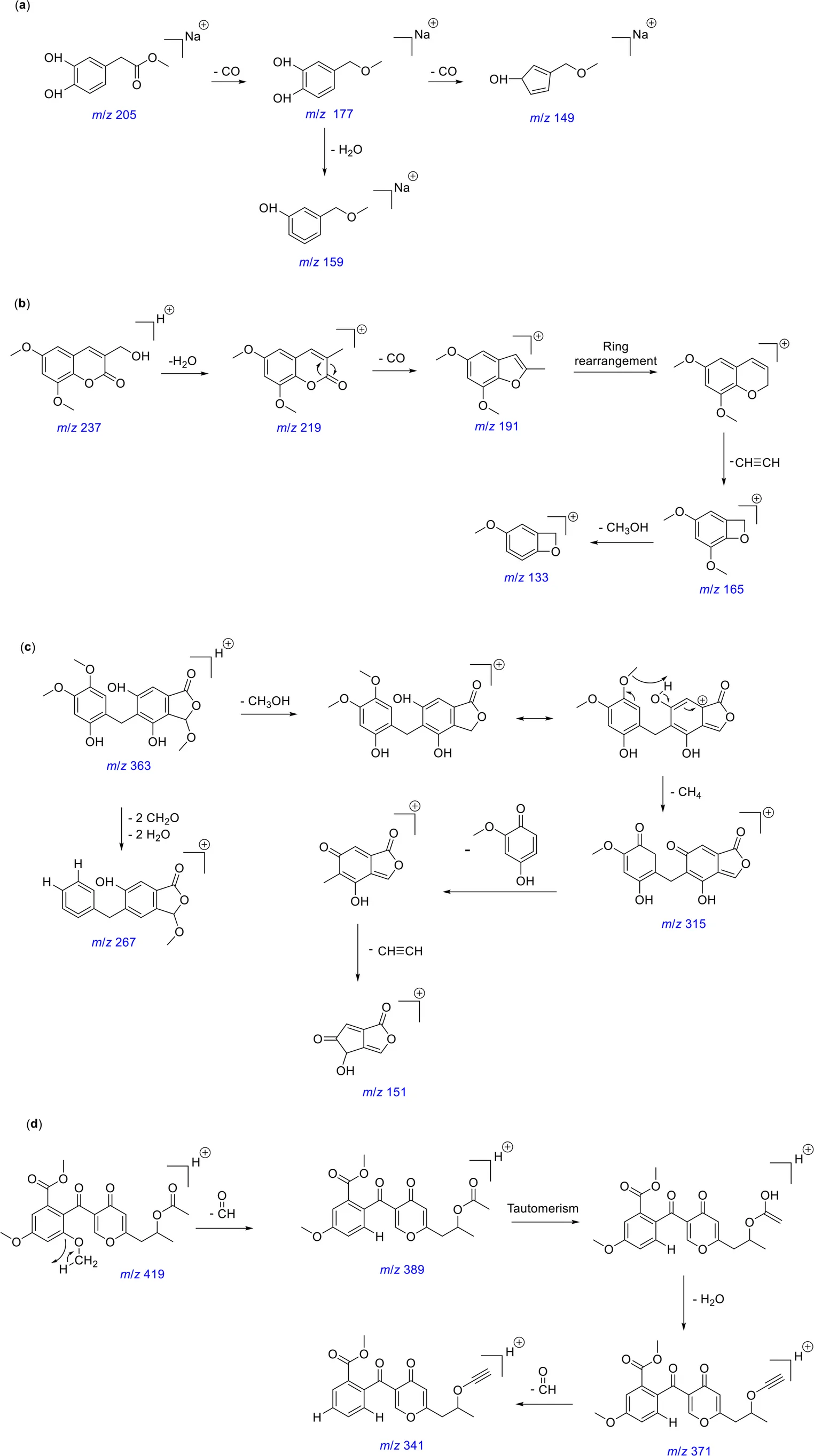
Proposed fragmentation pathway of 3,4-dihydroxyphenylacetic acid methyl ester (**a**, *m*/*z* 205), 3-(hydroxymethyl)-6,8-dimethoxy-2*H*-chromen-2-one (**b**, *m*/*z* 237), talaromycolide A (**c**, *m*/*z* 363), and actofunicone (**d**, *m*/*z* 419)

**Fig. 6:**
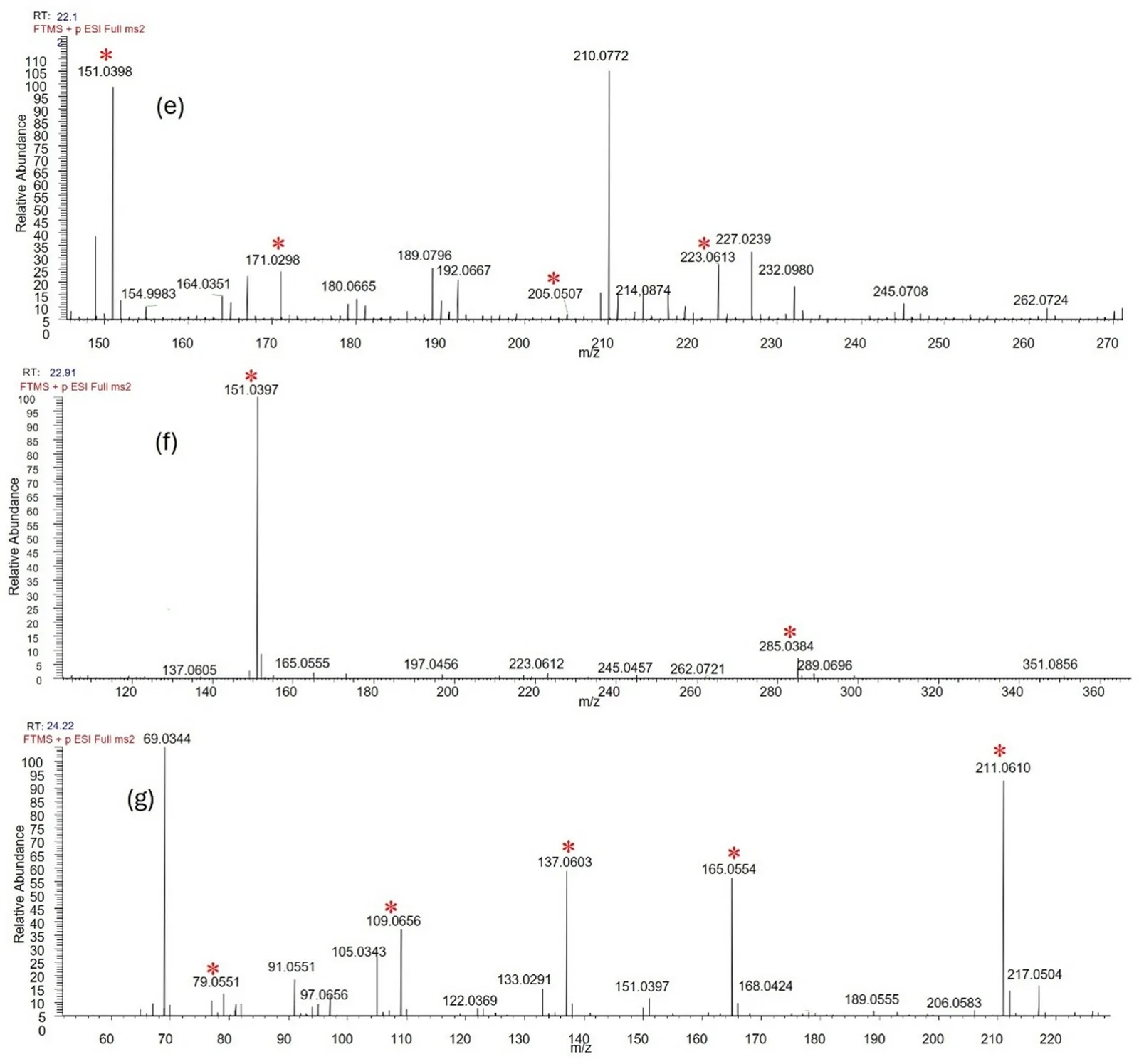
MS2 spectrum acquired in positive ion mode and proposed fragmentation pathway of amestolkin A (**e**), funicone (**f**), and rubralide C (**g**). The diagnostic fragment ions proposed in the fragmentation scheme (Fig. 7) are highlighted in the spectrum with red asterisks

**Fig. 7:**
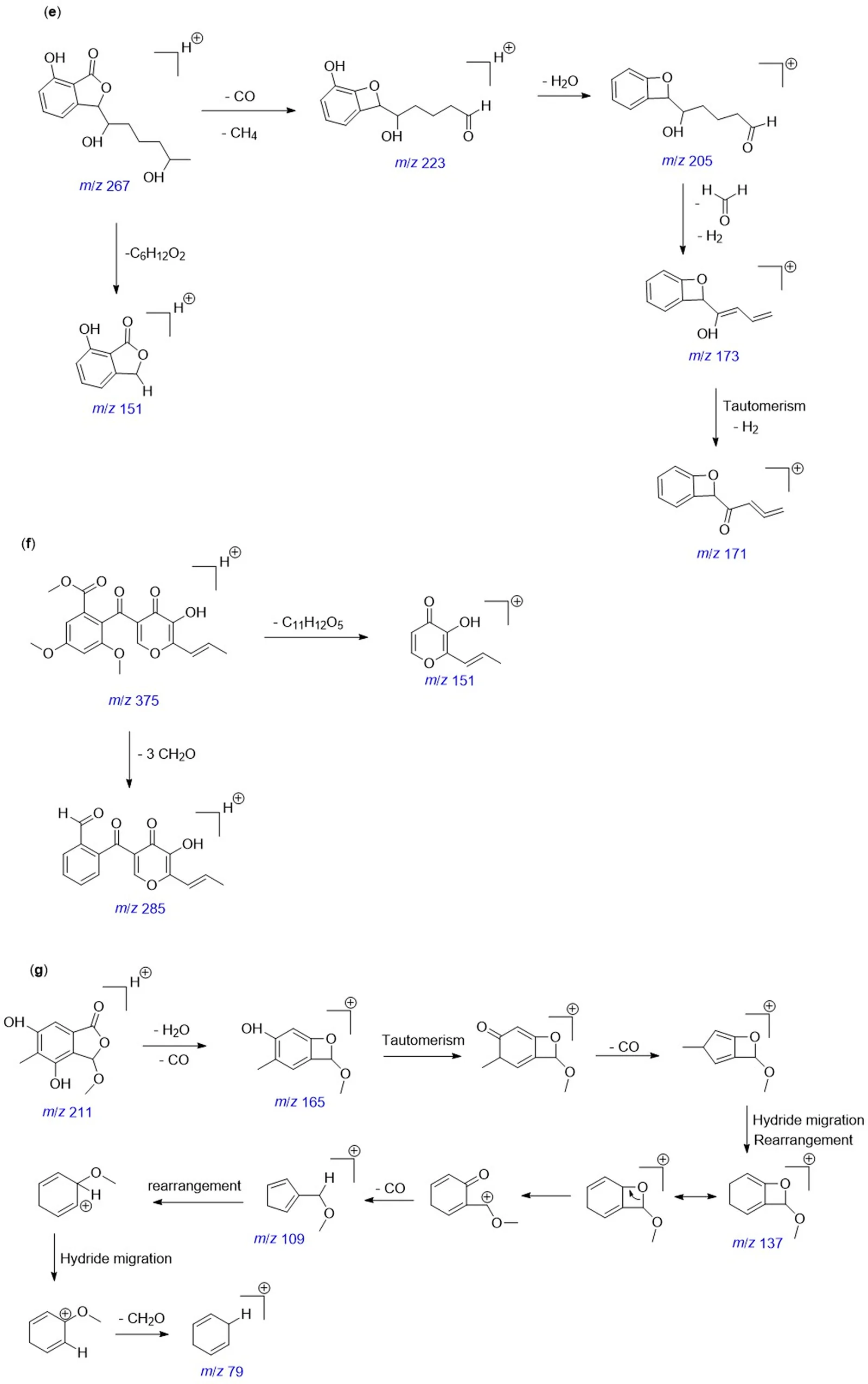
Proposed fragmentation pathway of 3,4-dihydroxyphenylacetic acid methyl esteramestolkin A (**e**, *m*/*z* 267), funicone (**f**, *m*/*z* 375), and rubralide C (**g**, *m*/*z* 211)

The MS2 spectra corresponding to compounds **a**–**g** (Figs. 4 and 6) were subsequently examined in detail, and tentative fragmentation pathways (Figs. 5 and 7) were proposed based on diagnostic fragment ions and characteristic neutral losses. The evidence supporting the putative annotation of these metabolites is summarized in Table 1, whereas the corresponding MS1 spectra are shown in Fig. S3 (Supplementary Material).

**Table 1:** Specialized metabolites exclusively annotated in the ethyl acetate extract of endophytic *Talaromyces pinophilus* J6 grown on PDA_AS based on UHPLC-ESI-QTOF-MS analysis and dereplication using a curated in-house database

ID	Retention time (min)	m/z, molecular formula, adduct	Δ m/z (ppm)	Putative identification (match with in-house database)
a	18.74	205.0460, C_9_H_10_O_4_, [M + Na]^+^	-5.36	3,4-dihydroxyphenylacetic acid methyl ester
b	20.72	237.0770, C_12_H_12_O_5_, [M + H]^+^	5.48	3-(hydroxymethyl)-6,8-dimethoxy-2*H*-chromen-2-one
c	21.51	363.1090, C_18_H_18_O_8_, [M + H]^+^	4.40	talaromycolide A
d	22.02	419.1350, C_21_H_22_O_9_, [M + H]^+^	3.10	actofunicone
e	22.13	267.1220, C_14_H_18_O_5_, [M + H]^+^	-2.62	amestolkin A
f	22.88	375.1090, C_19_H_18_O_8_, [M + H]^+^	4.26	funicone
g	24.67	211.0610, C_10_H_10_O_5_, [M + H]^+^	4.26	rubralide C

The MS1 spectrum of 3,4-dihydroxyphenylacetic acid methyl ester (**a**) showed a peak at *m*/*z* 205.0460 [M + Na]^+^, consistent with the molecular formula C_9_H_10_O_4_. This ion was also observed in its MS2 spectrum (Fig. 4a), where it generated a fragment at *m*/*z* 177 via CO loss. Subsequently, ions at *m*/*z* 149 (base peak) and 159 were formed from the *m*/*z* 177 ion through CO loss or dehydration, respectively (Fig. 5a). The coumarin 3-(hydroxymethyl)-6,8-dimethoxy-2 H-chromen-2-one (**b**) was detected at *m*/*z* 237.0770 in the MS1 spectrum, consistent with the calculated [M + H]^+^ ion for the molecular formula C_12_H_12_O_5_. The compound underwent extensive fragmentation, generating several product ions in the MS2 spectrum (Fig. 4b). Initial dehydration of the protonated ion yielded the fragment at *m*/*z* 219, which further produced the ion at *m*/*z* 191 after CO loss. Ring rearrangement and C_2_H_2_ loss from *m*/*z* 191 led to the formation of the fragment at *m*/*z* 165. Finally, the neutral loss of methanol from *m/z* 165 produced the ion at *m/z* 133 (Fig. 5b). Talaromycolide A (**c**) exhibited a base peak at *m*/*z* 363.1090 ([M + H]^+^, C_18_H_18_O_8_) in the MS1 spectrum. In the MS2 spectrum (Fig. 4c), the fragment ion at *m*/*z* 315 could be formed from *m*/*z* 363 through two sequential neutral losses of methanol and methane. Further fragmentation of the *m*/*z* 315 ion, involving cleavage of the linkage between the phenyl and phthalide moieties, followed by acetylene loss, led to the formation of the fragment at *m*/*z* 151. In addition, the ion at *m*/*z* 267 was generated directly from *m*/*z* 363 via successive losses of formaldehyde and water (Fig. 5c). The ion at *m*/*z* 419.1350 [M + H]^+^ was attributed to actofunicone (**d**). The fragmentation pathway of **d** was proposed based on its MS2 spectrum (Fig. 4d). Initially, the fragment at *m*/*z* 389 was formed via CHO loss from the precursor ion. Subsequent tautomerization of the *m*/*z* 389, followed by water loss, yielded the fragment at the *m*/*z* 371. Finally, *m*/*z* 371 ion fragmented to produce the ion at *m*/*z* 341 after formaldehyde loss (Fig. 5d).

The MS1 spectrum of the phthalide amestolkin A (**e**) displayed a peak at *m*/*z* 267.1220 [M + H]^+^, consistent with the molecular formula C_14_H_18_O_5_. In its MS2 spectrum (Fig. 6e), formation of the fragment at *m*/*z* 151 was proposed to result from cleavage of the side chain. An alternative fragmentation pathway, in which the side chain is retained, and the five-membered lactone ring of the phthalide nucleus undergoes contraction, was proposed to account for the ion at *m*/*z* 223. Subsequent sequential neutral losses from the *m/z* 223 ion gave rise to the fragments at *m/z* 205, 173, and 171 (Fig. 7e). Funicone (**f**, C_19_H_18_O_7_) was annotated at *m*/*z* 375.1090 [M + H]^+^. Its MS2 spectrum (Fig. 6f) was dominated by a single base peak at *m*/*z* 151, which was attributed to the formation of an ion resulting from the cleavage of the ketone bridge in the core structure (Fig. 7f). Rubralide C (**g**) was annotated from the protonated molecular ion at *m*/*z* 211.0610 [M + H]^+^, consistent with the molecular formula C_10_H_10_O_5_. Such an ion was also observed in its MS2 spectrum (Fig. 6g), allowing a comprehensive proposal of its fragmentation pathway. In this context, fragmentation of the *m*/*z* 211 ion began with successive losses of CO and H_2_O, leading to contraction of the five-membered lactone ring of the phthalide nucleus and formation of the fragment at *m/z* 165. After tautomerism and CO loss from *m*/*z* 165, the ion at *m*/*z* 137 was formed. Further CO loss and hydride migration from the *m*/*z* 137 ion account for the formation of the fragments at *m*/*z* 109 and 79, respectively (Fig. 7g).

### Isolation and identification of the main specialized metabolites from *T. pinophilus* J6

Based on the chemical potential of *T. pinophilus* J6 and its distinctive metabolic profile in PAD_AS medium, this strain was cultivated on a large scale to enable the isolation of its main specialized metabolites (highlighted with an asterisk in the BPC of Fig. 3). The isolated compounds **1** and **2** were detected at retention times of 24.48 and 24.44 min, respectively. The corresponding BPC peak areas were 1E6 for compound **1** and 8E8 for compound **2**.

The resulting scale-up crude extract was fractionated by column chromatography and, to further enhance the purities, the isolated compounds were eluted from SPE cartridges prior to 1D and 2D NMR spectroscopic analyses. The NMR and HRESIMS data (Figs. S4-S15, Supplementary Material) allowed the structural elucidation of the new (-)-*R*-talaropinophiloic acid (**1**) and the identification of the 3-*O*-methylfunicone (**2**), whose chemical structures are depicted in Fig. 8.

**Fig. 8:**
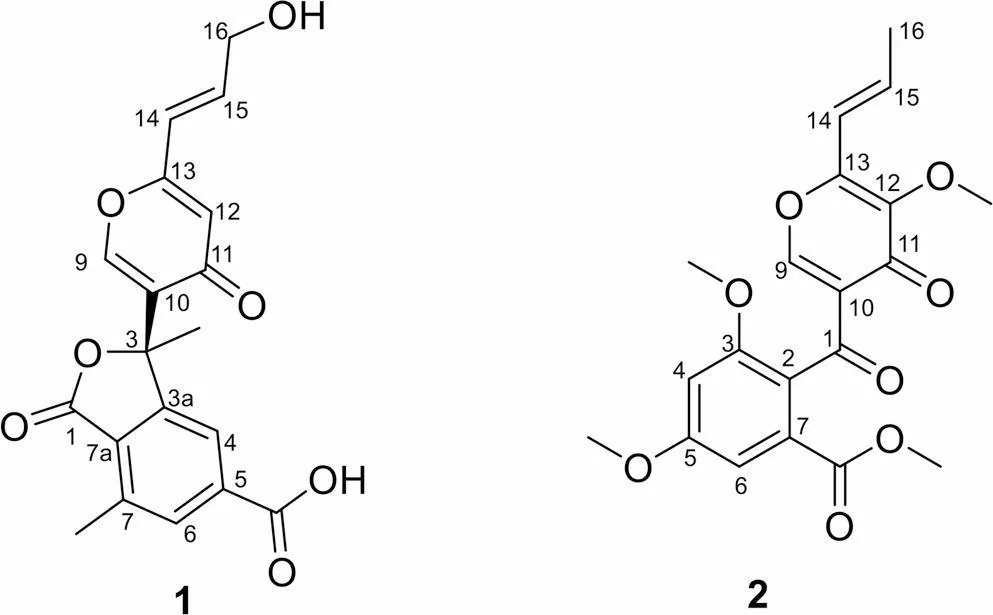
Chemical structures of (-)-*R*-talaropinophiloic acid (**1**) and 3-*O*-methylfunicone (**2**) isolated from the *Talaromyces pinophilus* J6 grown in PDA medium supplemented with ammonium sulfate

The molecular formula of the minor purified compound (**1**) was determined as C_19_H_16_O_7_, deduced from HRESIMS at *m/z* 357.0983 [M + H]^+^ (Fig. S10), and its complete NMR spectroscopic data (Figures S4-S9) are summarized in Table 2. The ^1^H NMR spectrum (Fig. S4) showed two singlet methyl groups at *δ*_H_ 1.65 and 2.60, one oxymethylene group at *δ*_H_ 4.30 (*dd*), and six hydrogens attached to distinct Csp^2^-hybridized at *δ*_H_ 6.14 (*d*), 6.26 (*d*), 6.43 (*d*), 6.58 (*s*), 6.74 (*dt*), and 8.19 (*s*). The coupling constant of *J* 16.0 Hz observed for both doublet H-14 (*δ*_H_ 6.43) and doublet of triplet H-15 (*δ*_H_ 6.74) indicated the *trans* configuration of the allylic double bond between C-14 and C-15. Additionally, the two aromatic hydrogen (*δ*_H_ 6.14 and 6.26) were assigned as meta-coupled based on their multiplicity (doublets) and coupling constants of *J* 2.5 Hz.

The ^13^C NMR spectrum (Fig. S5) displayed 19 carbon signals. Three signals in the *δ*_C_ 145–146 range, which may be confirmed by expansion in Fig. S5, were attributed to sp^2^ carbons at positions C-3a (*δ*_C_ 145.2), C-7 (*δ*_C_ 145.8), and C-10 (*δ*_C_ 145.3). In addition, the combined analysis of ^13^C NMR and HSQC data revealed sp^2^ carbons lacking directly attached hydrogens (quaternaries): *δ*_C_ 164.6 (C-5), 105.0 (7a), 156.1 (C-9), 110.8 (C-12), 157.6 (C-13), 120.8 (C-14), 140.3 (C-15), and 86.5 (C-3). The chemical shifts of C-3a and C-7a are consistent with those previously reported for similar natural products (Habib et al. 2008). The hydrogenated aromatic carbons were established as *δ*_C_ 101.7 (C-4) and 112.8 (C-6). Two methyl groups at *δ*_C_ 22.8 (attached to C-3) and 24.1 (attached to C-7), along with one oxymethylene carbon at *δ*_C_ 62.4 (C-16), were also assigned. The most deshielded carbons *δ*_C_ 194.0, 171.1, and 166.4 were attributed to carbonyl groups at C-11, C1, and C-5-COOH, respectively.

Detailed analysis of HMBC (Fig. S7), TOCSY (Fig. S8), and COSY (Fig. S9) spectra confirmed the proposed structure of compound **1**. The methyl group at *δ*_H_ 2.60 was placed linked to C-7 based on HMBC correlation with aromatic quaternary carbons at *δ*_C_ 145.8 (C-7), 112.8 (C-6), and 105.0 (C-7a) as well as a weak correlation with the carbonyl group at C-1 (*δ*_C_ 171.7). Additionally, multiple HMBC correlations between H-6 (*δ*_H_ 6.26) and C-4, C-5, C-7a, or 7-CH_3,_ together with correlations between H-4 (*δ*_H_ 6.14) and C-5, C-6, C-7a or 5-COOH established the phthalide moiety. The attachment of the methyl group at *δ*_C_ 22.8 to C-3 (*δ*_C_ 86.5) generated an unreported asymmetric center at this position and was supported by HMBC correlation between its hydrogens (*δ*_H_ 1.65) and C-3. The furanone ring was also connected to C-3, as indicated by HMBC correlations between the 3-CH_3_ hydrogens and C-11 (*δ*_C_ 194.0). The proposition of this ring was further supported by correlations from both H-9 (*δ*_H_ 8.19) and H-12 (*δ*_H_ 6.58) to C-10, C-11, and C-13. The allylic alcohol side chain was identified based on long-range HMBC correlations of H-14 (*δ*_H_ 6.43) with C-13, C-15, or C-16, and of H-15 (*δ*_H_ 6.74) with C-13 or C-16. The correlation between H-12 (*δ*_H_ 6.58) and C-14 indicated a π-extended system in this structure region and unequivocally confirmed the attachment of allylic alcohol at C-13. The primary alchool at C-16 (*δ*_H_ 62.4) was confirmed by HMBC correlations between H-16 (*δ*_H_ 4.29) and C-14 or C-15. Finally, TOCSY analysis supported the assignment of the aromatic methyl group (at C-7) through its correlation with H-6 and also confirmed the connectivity of the allylic side chain.

**Table 2:**
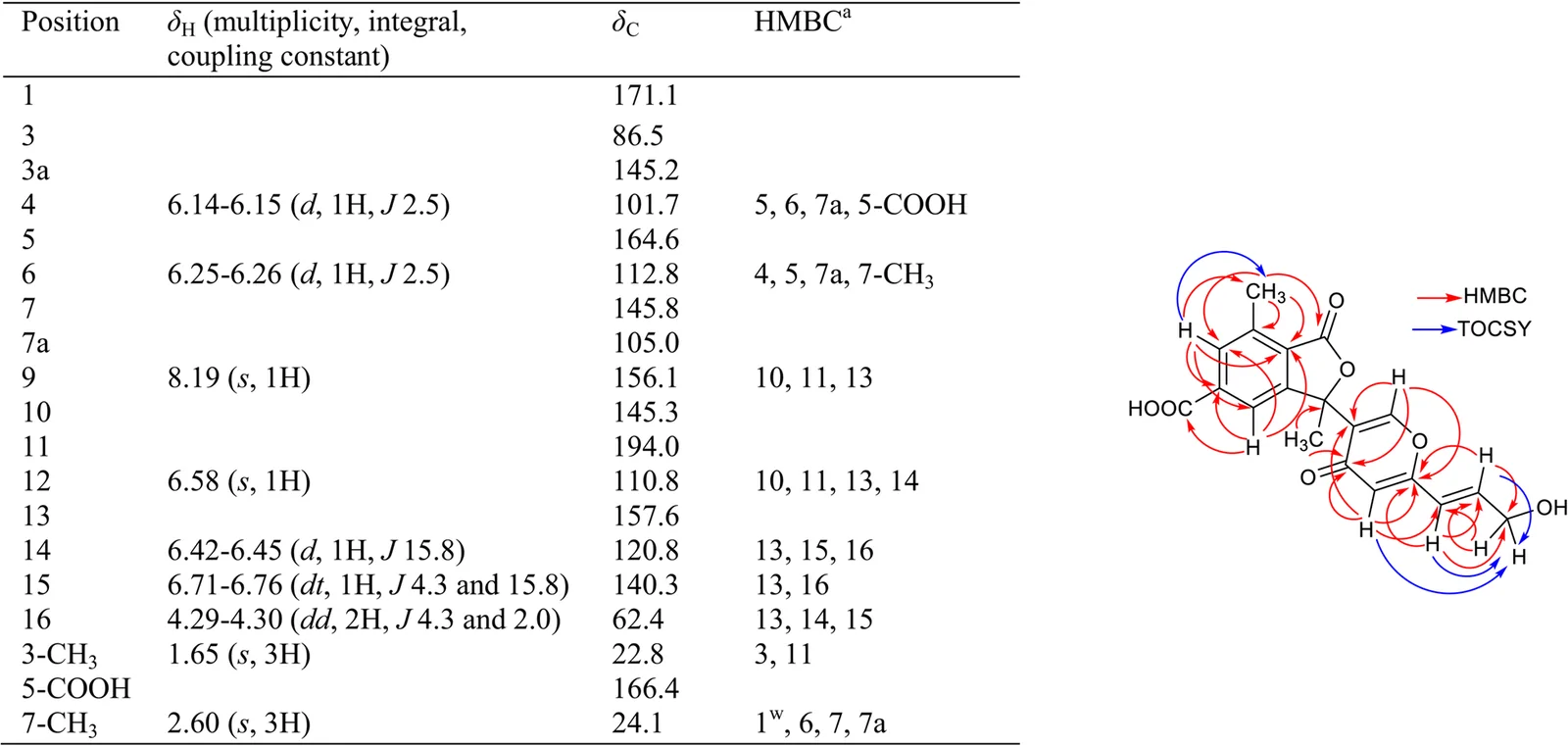
^1^H and ^13^C NMR data (500 and 125 MHz, respectively), HMBC assignment of (-)-*R*-talaropinophiloic acid in CD_3_OD (*δ* in ppm, *J* in Hz)

^a^HMBC correlations, optimized for 6 Hz, are from hydrogen stated to the indicated carbon. ^w^weak correlation

**Fig. 9:**
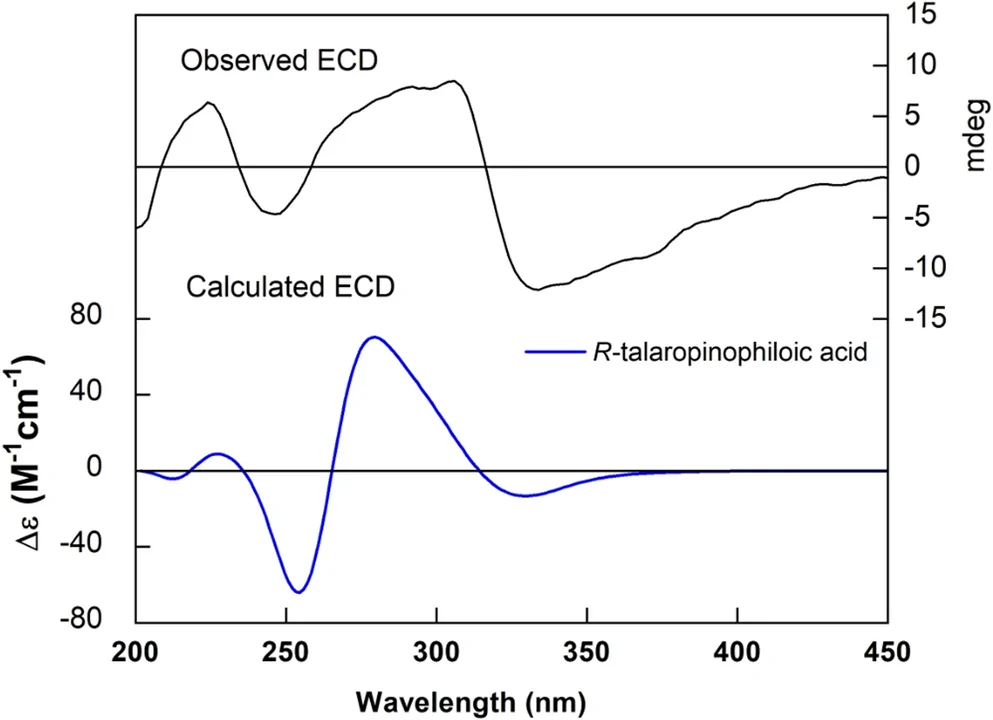
Experimental and calculated ECD spectra for (-)-*R*-talaropinophiloic acid (**1**)

The absolute configuration of the asymmetric center at C-3 in compound **1** was determined by comparing the experimental and calculated ECD data (Fig. 9). The good agreement observed between the experimental and simulated spectra allowed the unequivocal assignment of the absolute configuration of (-)-**1** as 3*R*. Therefore, the trivial name (-)-*R*-talaropinophiloic acid was subsequently given for compound **1**.

Finally, the major isolated compound presented a protonated ion at *m*/*z* 389.1244 in the HRMS spectrum (Fig. S15), consistent with the molecular formula C_20_H_20_O_8_. The 1D and 2D NMR spectroscopic data (Figs. S11–S14) allowed its unequivocal structural identification as 3-*O-*methylfunicone (De Stefano et al. 1999). Briefly, the singlets at *δ*_H_ 3.88, 3.78, 3.75, and 3.73 were attributed, respectively, to methoxy groups attached at positions 5, 7, 12, and 3. The two ethylenic hydrogens (*δ*_H_ 6.61 and 6.74, at C-14 and C-15, respectively) were in *E* configuration because of the mutual coupling constant (*J* 15.0 Hz). Moreover, the methyl group at *δ*_H_ 1.97 (C-16), was attached at C-15 based on its correlations with C-14 (*δ*_C_ 119.3) and C-15 (*δ*_C_ 137.4), suggesting a propenyl side chain. The aromatic H-6 and H-4 (*δ*_H_ 7.07 and 6.81) had a meta coupling constant (*J* 2.0 Hz). Signals at *δ*_C_ 174.3 (C-11), 161.5 (C-9), 156.8 (C-13), 145.2 (C-12), and 128.0 (C-10) in ^13^C NMR, along with the 2D correlations with the methine hydrogen *δ*_H_ 8.48 (position 9), were in good agreement for a *γ*-pyrone nucleus.

### Cytotoxic activity

Given the previously reported cytotoxic activity of latex and extracts from *E. umbellata*, we investigated the in vitro cytotoxic potential of the two fungal-derived polyketides (–)-*R*-talaropinophiloic acid (**1**) and 3-*O*-methylfunicone (**2**) against bladder cancer cells (T24). Their inhibitory effects were also assessed in normal cell lines (RPE-1) to evaluate selectivity.

**Fig. 10:**
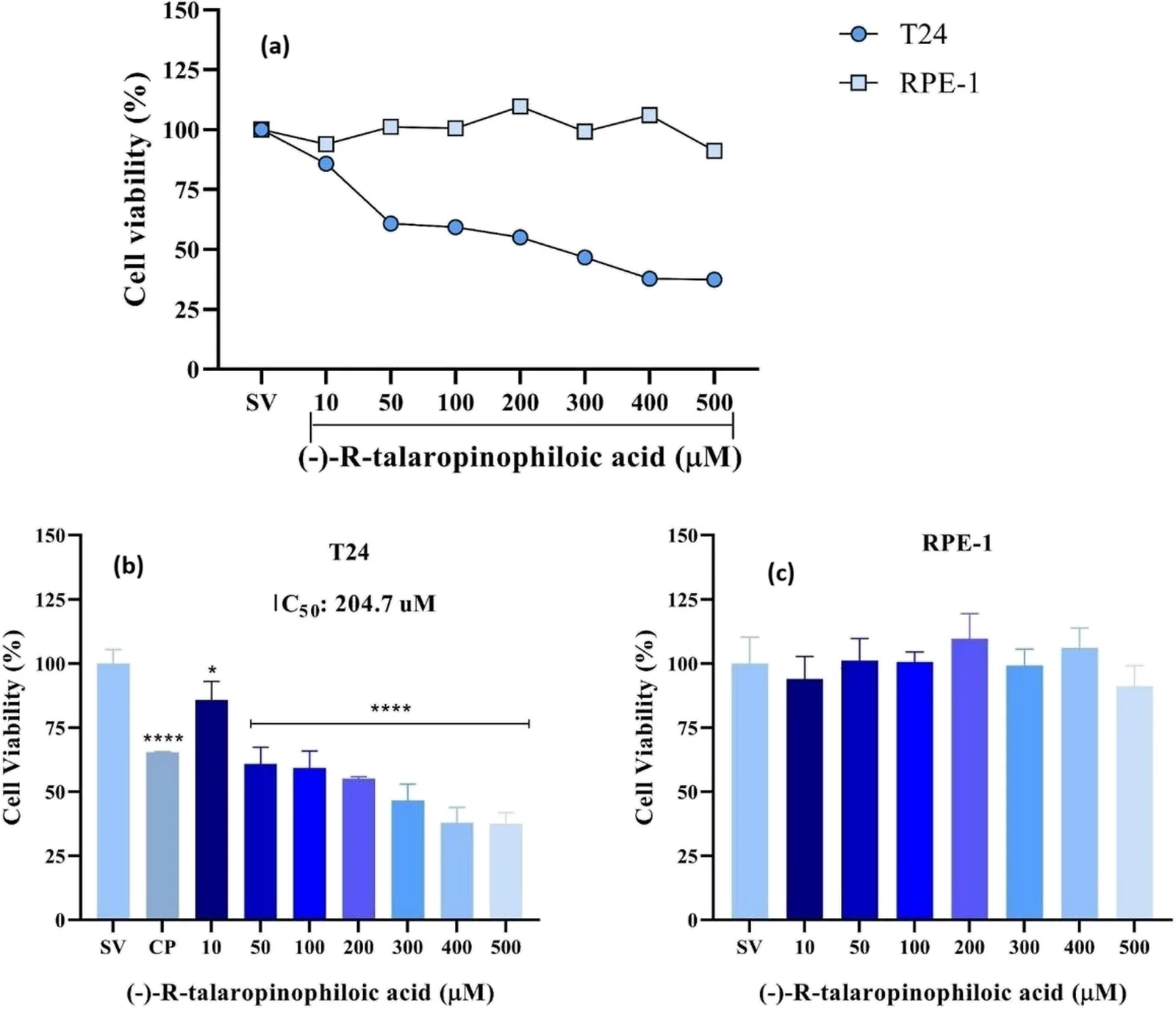
Cytotoxic activities (MTT assay) displayed by (-)-*R*-talaropinophiloic acid: (**a**) the viability fraction (%) of bladder cancer T24 cells and normal RPE-1 cell lines after treatment with (-)-*R*-talaropinophiloic acid (10, 50, 100, 200, 300, 400, and 500 µM). Cisplatin was the positive control (6.4 µM); (**b**) IC_50_ displayed by (-)-*R*-talaropinophiloic acid on T24 cells.; (**c**) effect of (-)-*R*-talaropinophiloic acid on RPE-1 cells. All values are presented as the mean ± standard deviation (X ± SD). Values statistically different from SV (solvent vehicle, DMSO 0.25%) at the respective time point (*p < 0.05; ****p < 0.0001 ANOVA followed by Dunnett’s post-test)

Compound **1** displayed selective cytotoxicity toward the tumor cell line (Fig. 10). In T24 cells, **1** displayed an IC_50_ value of 204.7 µM, indicating weak cytotoxicity. In contrast, no CC_50_ value was determined for the normal RPE-1 cells, as compound **1** did not significantly reduce cell viability within the tested concentration range.

**Fig. 11:**
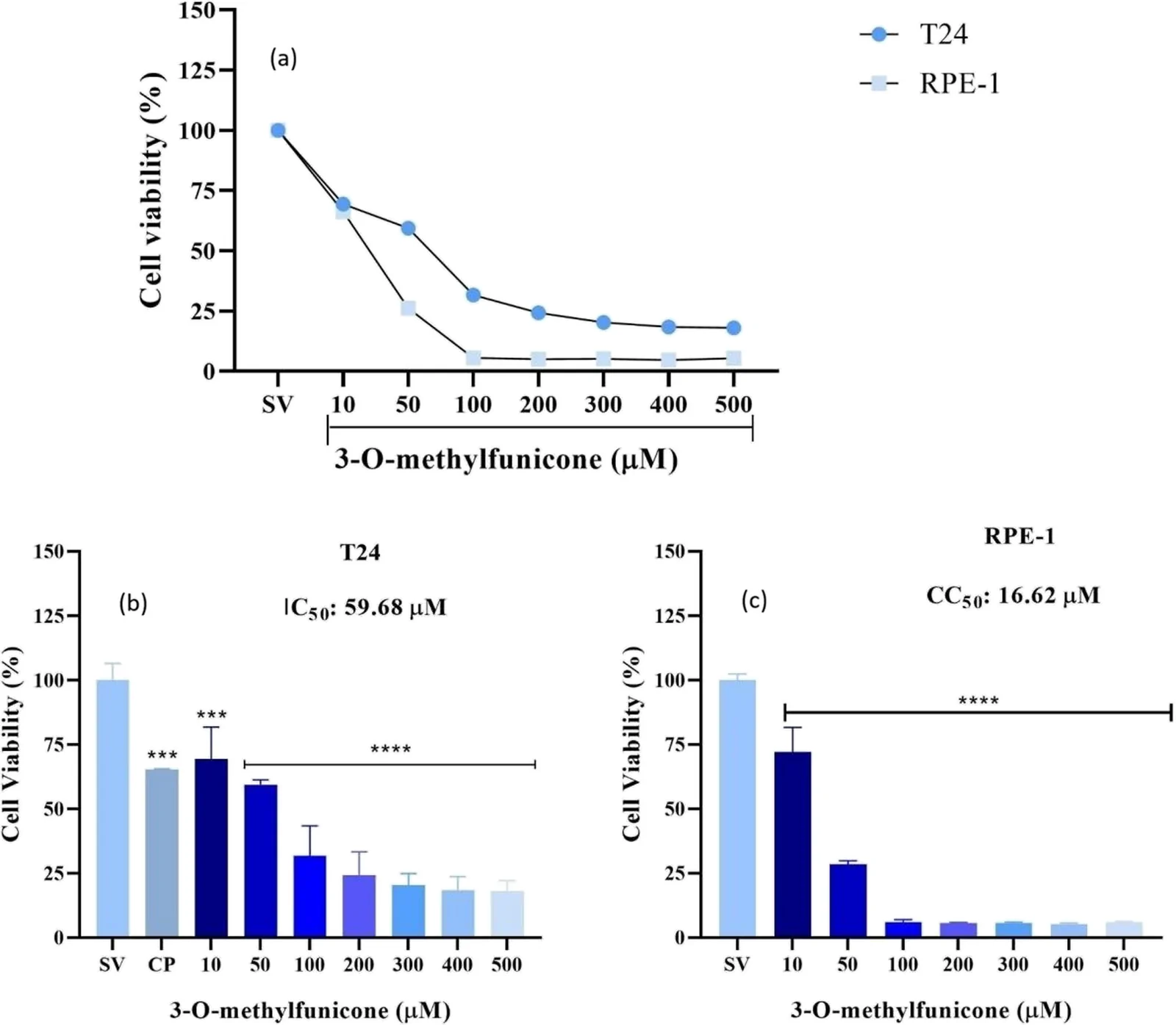
Cytotoxic activities (MTT assay) displayed by 3-*O*-methylfunicone: (**a**) the viability fraction (%) of bladder cancer T24 cells and normal RPE-1 cell lines after treatment with 3-*O*-methylfunicone (10, 50, 100, 200, 300, 400, and 500 µM). Cisplatin was the positive control (6.4 µM); (**b**) IC_50_ displayed by 3-*O*-methylfunicone on T24 cells.; (**c**) CC_50_ of 3-*O*-methylfunicone on RPE-1 cells. All values are presented as the mean ± standard deviation (X ± SD). Values statistically different from SV (solvent vehicle, DMSO 0.25%) at the respective time point (*p < 0.05; ****p < 0.0001 ANOVA followed by Dunnett’s post-test)

On the other hand, compound **2** exhibited higher overall cytotoxicity across both evaluated cell lines (Fig. 11). In T24 cells, compound **2** exhibited an IC_50_ value of 59.68 µM, markedly lower than that observed for compound **1**, confirming its higher cytotoxic activity. However, compound **2** also substantially reduced the viability of normal RPE-1 cells, with a CC_50_ value of 16.62 µM (SI 0.27).

## Discussion

### Chemical comparisons and selection of the endophyte *T. pinophilus* J6

The endophytes J1, J5, J6, J7, J9, and J13, isolated from *Euphorbia umbellata*, were grown in Petri dishes containing PDA or PDA supplemented with inorganic salts (sodium tartrate dihydrate or ammonium sulfate). Modifications in the composition of culture media by incorporating inorganic salts have been reported as an interesting trigger factor to produce new specialized metabolites by fungi (Ariantari et al. 2019). Thus, sodium tartrate dihydrate and ammonium sulfate were investigated as potential agents capable of altering individual chemical responses of endophytes, thereby enabling the biosynthesis of new chemical entities.

Untargeted metabolomic analysis based on UHPLC/ESI Q-Orbitrap data was performed to compare ethyl acetate extracts from all endophyte cultures. The BPC (Figure S1) analysis provided a visual overview of the chemical complexity of the samples, supporting the subsequent feature extraction and multivariate analyses. The BPCs exhibited stable baselines and well-resolved peaks, confirming the high quality of the LC-HRMS acquisition.

Next, an unsupervised multivariate data analysis using PCA (Fig. 1) enabled visualization of sample grouping and data dimensionality reduction. PCA is a valuable multivariate tool for data exploration and experimental planning, as it enables the visualization of chemical differences among sample classes in a reduced-dimensional space. The analysis focused on comparing chemical profiles of endophyte strains grown in distinct culture media. We acknowledge that the inclusion of internal standards and pooled quality control samples (QC) would further strengthen the robustness of the metabolomic dataset. However, because the study focused on detecting relative differences in metabolic profiles among fungal strains rather than obtaining absolute or semi-quantitative measurements, the resulting data was deemed adequate for prioritization. PCA analysis showed that, among all analyzed samples, the crude extracts of strain J6 grown on PDA and PDA_AS were clearly separated from those of the other fungi. Notably, the J6 strain extract grown on PDA_AS medium showed the greatest separation in PCA, indicating a unique metabolite profile compared with the other samples. This marked chemical distinction suggests enhanced metabolic diversity and/or the induction of novel specialized metabolites under PDA_AS conditions. Therefore, cultivation of J6 on PDA_AS was prioritized for further chemical investigation to discover new natural products.

Strain J6 was identified as *Talaromyces pinophilus* by molecular and phylogenetic analyses of the benA gene (Fig. 2). The genus *Talaromyces* (Ascomycota) has attracted increasing scientific interest, as it is recognized as a prolific source of structurally diverse specialized metabolites exhibiting a wide range of biological activities (Lei et al. 2022). Although *Talaromyces* spp. have been classically associated with soil and food substrates, some species have also been identified as endophytes, inhabiting different plant hosts (Zhang et al. 2024a). In this ecological niche, they adjust their metabolic pathways in response to the plant environment, resulting in the production of compounds with antifungal, antibacterial, and antioxidant (da Silva et al. 2017).

Recent advances in the study of specialized metabolites from the *Talaromyces* genus have been reported. In addition to the growing interest in their specialized metabolites, endophytic *Talaromyces* species have been associated with diverse biological applications, including the biosynthesis of extracellular silver nanoparticles for therapeutic purposes by *T. funiculosus* (El deeb et al. 2025) and the promotion of plant growth through root colonization by *T. albobiverticillius* (Kharkwal et al. 2024). Given the increasing recognition of the biotechnological relevance of this genus, the identified endophytic strain *T. pinophilus* J6 was subsequently subjected to comprehensive chemical characterization.

### Dereplication approach to profile *T. pinophilus* J6 specialized metabolites

Natural products from endophytic fungi are highly attractive as drug leads because they occupy a broader chemical space and exhibit greater drug-like properties than compounds derived from combinatorial chemistry (Samanta et al. 2021). However, discovering new natural products remains challenging. Even when an organism’s metabolome is successfully stimulated, novel specialized metabolites are often produced in low abundance and are masked by known compounds present at higher concentrations (Tabudravu et al. 2019). Therefore, it is imperative to implement strategies that enable rapid identification of known metabolites at an early stage, thereby allowing research efforts to focus on the discovery of structurally novel compounds.

Accordingly, exploratory strategies, such as UHPLC–HRMS-based dereplication, have been increasingly adopted in natural products research for comprehensive chemical characterization (Nielsen and Larsen 2015). Early chemical dereplication plays a critical role by facilitating the efficient detection and annotation of known metabolites while guiding the achievement of previously unreported specialized metabolites. Dereplication using in-house databases typically involves dedicated data evaluation and often leads to rapid, successful putative identification of known compounds (De Medeiros et al. 2015a).

In our ongoing efforts to understand the specialized metabolic profiles of endophytic fungi (Ribeiro et al. 2021; Gusmão et al. 2024), we report herein the metabolic profiling of *T. pinophilus* J6 using UHPLC/ESI Q-Orbitrap data analysis, combined with queries of the in-house *Talaromyces* sp. database. Owing to the exclusive chemical profile exhibited in the BPC from the investigated crude extracts, *T. pinophilus* J6 grown in PDA_AS medium was chosen as the focus of the study, and likewise for the results discussed hereafter.

Detailed analysis of the BPC of *T. pinophilus* J6 grown in PDA_AS medium showed that most peaks were between 18 and 26 min of retention time (Fig. 3). A dereplication workflow was applied using a curated in-house database comprising 773 metabolites previously reported for the genus *Talaromyces*. Dereplication was performed by integrating the in-house library directly into the MZmine platform. The putative identification was initially carried out based on the MS1 analysis and matching the base peak with the in-house database. After this, MS2 spectra were carefully inspected, and fragmentation pathways were proposed to improve annotation levels. Our dereplication strategy led to the annotation of seven natural products: 3,4-dihydroxyphenylacetic acid methyl ester (**a**), 3-(hydroxymethyl)-6,8-dimethoxy-2*H*-chromen-2-one (**b**), talaromycolide A (**c**), actofunicone (**d**), amestolkin (**e**), funicone (**f**), rubralide C (**g**). Compounds **a**-**g** were exclusively detected in the PDA_AS medium. Table S1 highlights the peak area corresponding to each of the **a**-**g** prioritized compounds. Overall, the combination of accurate mass measurements and detailed MS2 fragmentation analyses enabled the confident annotation of seven specialized metabolites (**a**-**g**) exclusively produced by *T. pinophilus* J6 in PDA_AS medium. The putatively identified compounds belong mainly to coumarin, phthalide, and funicone-related classes, many of which are known for their biological activities.

All natural products **a**-**g** dereplicated in the extract of *T. pinophilus* J6 have previously been reported in other *Talaromyces* strains. The compound **a** was isolated from *Talaromyces assiutensis* JTY2 cultures and displayed antifungal activity against phytopathogenic fungi (Li et al. 2022). The coumarin **b** has previously been isolated from *Talaromyces flavus* (Zhai et al. 2016). Talaromycolide A (**c**) was previously isolated from *T. pinophilus* AF-02. It is a rare phthalide derivative, and it has been reported to exhibit significant antibacterial activity against *Bacillus subtilis*, *B. megaterium*, *Escherichia coli*, *Clostridium perfringens*, and *Micrococcus tetragenus* (Zhai et al. 2015). Actofunicone (**d**) was previously reported from the soil-derived strain *T. flavus* FKI-0076 (Zhai et al. 2016). The phthalide amestolkin A (**e**) has previously been isolated from cultures of *Talaromyces amestolkiae* and was reported to inhibit the expression of pro-inflammatory factors (Huang et al. 2023). Funicone (**f**) and its analogs have been widely reported within the *Talaromyces* genus and frequently exhibit inhibitory activity against pathogenic fungi (Zhai et al. 2016). Rubralide C (**g**) is a phthalide derivative previously reported from *T. pinophilus* AF-02 (Zhai et al. 2015).

Finally, the main peak in the BPC (highlighted with an asterisk in Fig. 3) deserves attention not only because of its high intensity, but also due to the number of natural products that co-eluted within the same retention time range (24.0–24.5 min). According to Table S1 (Supplementary material), eight compounds were detected in this interval, including potential unknown compounds. Among these metabolites, 3-*O*-methylfunicone was annotated, but it was not included in the dereplication study, as our focus was on the specialized metabolites produced exclusively by *T. pinophilus* J6 when grown on PDA_AS. As previously reported, 3-*O*-methylfunicone is produced by *T. pinophilus* J6 under different culture conditions (Santos et al. 2025; Pereira et al. 2026).

### Isolation and unequivocal identification of the main specialized metabolites of *T. pinophilus* J6 and their cytotoxic activities

Continuing our efforts to achieve pure specialized metabolites from endophytic fungi, *T. pinophilus* J6 was cultivated on a large scale in PDA_AS medium. The scale-up fermentation crude extract was subjected to chromatographic purification to isolate specialized metabolites corresponding to the main peak in the BPC, highlighted with an asterisk in Fig. 3. Two specialized metabolites were isolated using the applied chromatographic protocol. Analysis of NMR and HRESIMS data enabled the structural elucidation of the new compound (-)-*R*-talaropinophiloic acid (**1**) and the identification of 3-*O*-methylfunicone (**2**).

The ^1^H and ^13^C NMR data of **1** were similar to those of the known vermistatin (Komai et al. 2004) and talaromurolide D (Zhu et al. 2024), suggesting a common scaffold. A notable difference in compound **1** is the presence of an asymmetric center at C-3 (*δ*_C_ 86.5) where a methyl group (*δ*_C_ 22.8) is attached, instead of a hydrogen, as observed in vermistatin and talaromurolide D. Additional structural features that distinguish compound **1** and support its classification as a previously unreported molecule include the substitution pattern on the aromatic ring. While both vermistatin and talaromurolide D possess methoxy substituents on their aromatic rings, compound **1** instead features methyl and carboxyl groups. The proposed structure 1 was fully supported by detailed analyses of their HMBC and TOCSY spectra. The unusually high chemical shift of the carbonyl carbon at C-11 (*δ*_C_ 194.0) may be attributed to hydrogen bonding between the carbonyl oxygen and methanol used as the NMR solvent. This hypothesis, however, requires further investigation for confirmation.

The absolute configuration of the asymmetric center at C-3 in compound **1** was determined by comparing the experimental and calculated ECD data. This methodology has been demonstrated to be a powerful and reliable tool for configurational analysis of complex natural products across different classes (Pescitelli and Bruhn 2016). The good agreement observed between the experimental and simulated spectra (Fig. 9) allowed the unequivocal assignment of the absolute configuration of (-)-**1** as 3*R*. Therefore, the trivial name (-)-*R*-talaropinophiloic acid was subsequently given for compound **1**. It is worth mentioning that the previously reported vermistatin scaffold corresponds to the 3*S* isomer.

Finally, NMR spectroscopic analysis of compound **2** unequivocally identified it as 3-*O-*methylfunicone (De Stefano et al. 1999). Funicone-related metabolites are fungal polyketides characterized by a γ-pyrone moiety connected to an α-resorcylic acid unit via a ketone linkage. These compounds are predominantly reported from species of the genus *Talaromyces* and exhibit diverse biological activities, including antitumor effects (Manzo and Ciavatta 2012; Marques et al. 2026). Initially recognized for their role in microbial antagonism, funicones and their analogs have since attracted significant attention for their notable bioactivity, positioning them as promising candidates for drug discovery (Salvatore et al. 2022).

Following the unequivocal chemical characterization of the pure compounds (–)-*R*-talaropinophiloic acid (**1**) and 3-*O*-methylfunicone (**2**) and given the previously reported cytotoxic activity of *E. umbellata*, we investigated the in vitro cytotoxic potential of these two fungal-derived polyketides against bladder cancer cells (T24). Their inhibitory effects were also assessed in normal cell lines (RPE-1) to evaluate selectivity.

Compound **1** displayed selective cytotoxicity toward the tumor cell line (Fig. 10). In T24 cells, it had an IC_50_ of 204.7 µM, indicating weak cytotoxicity. In contrast, no CC_50_ value was determined for the normal RPE-1 cells, as compound **1** did not significantly reduce cell viability within the tested concentration range. These findings suggest a favorable selectivity profile for compound **1** toward cancer cells. On the other hand, compound **2** showed higher overall cytotoxicity across both cell lines (Fig. 11). In T24 cells, it had an IC_50_ of 59.68 µM, markedly lower than that of compound **1**, confirming its greater cytotoxicity. However, compound **2** also substantially reduced the viability of normal RPE-1 cells, with a CC_50_ value of 16.62 µM, indicating reduced selectivity and increased general toxicity (SI 0.27).

Both isolated polyketides exhibited distinct cytotoxic profiles against bladder cancer cells. Compound **1** demonstrated weak cytotoxicity but favorable selectivity toward tumor cells, whereas compound **2** exhibited greater potency but reduced selectivity due to its marked toxicity toward normal cells. These results highlight that potency and selectivity must be considered together when assessing anticancer candidates. Although compound **1** was less potent, its preferential cytotoxicity toward cancer cells indicates a more favorable therapeutic profile. Importantly, compound **1** represents a previously unreported chemotype, expanding the chemical diversity of bioactive fungal polyketides. Its selective activity and comparatively low toxicity support its potential as a promising hit compound for future derivatization and lead optimization studies. Further investigations are warranted to elucidate its mechanism of action and to determine whether its biological activity can be enhanced through structural modification while maintaining its selectivity.

Previous reports have shown that 3-*O*‐methylfunicone (**2**) targets multiple tumorigenic pathways, underscoring its anticancer potential. In breast tumor cells, **2** decreased the expression of the survivin and telomerase activity, resulting in the death of breast tumor cells (MCF-7) (Buommino et al. 2011), and induced inhibition of cell motility through the modulation of integrin and MMP-9 secretion (Buommino et al. 2007). Additionally, **2** has also inhibited mesothelioma cell proliferation and, when combined with cisplatin, reduced migratory capacity through the downregulation of metalloproteinase-2 and suppressed angiogenesis by decreasing VEGF gene expression (Buommino et al. 2012). The 3‐*O*‐methylfunicone (**2**) inhibited melanoma (A375) cell proliferation in a time- and dose-dependent manner by inducing G2 phase arrest, modulating cell cycle regulators and survival pathways, and impairing cellular replicative capacity (Baroni et al. 2009).

## Conclusion

In summary, chemical investigation of the fermentation products of the fungal strain *T. pinophilus* J6 enabled a comprehensive characterization of its crude extract, resulting in the annotation of seven metabolites (**a**-**g**) and guiding the isolation of two polyketides: the new (-)-*R*-talaropinophiloic acid (**1**), along with the known compound 3-*O*-methylfunicone (**2**). The results highlight the effectiveness of metabolomics-assisted workflows for accelerating the preliminary identification of fungal specialized metabolites. Furthermore, cultivating *T. pinophilus* J6 under modified conditions enhanced the production of cryptic metabolites and led to the discovery of new natural products with relevant biological activities.

Both isolated polyketides exhibited distinct cytotoxic profiles against bladder cancer cells. Compound **1** exhibited weak cytotoxic activity with favorable selectivity for tumor cells, whereas compound **2** displayed higher potency but reduced selectivity due to its pronounced toxicity toward normal cells.

This is the first report describing the metabolomic investigation of the endophytic fungus *T. pinophilus* J6 isolated from *E. umbellata*, reinforcing the potential of endophytic fungi associated with medicinal plants as sources of chemically diverse and biologically relevant natural products. Integration of changes in fungal growth conditions and dereplication using an in-house database proved a successful approach for discovering a new natural product. Nevertheless, further mechanistic, biosynthetic, and in vivo studies are required to fully elucidate the biological potential of the isolated compounds.

## Supplementary Information

Below is the link to the electronic supplementary material.

Supplementary Material 1

## Data Availability

No datasets were generated or analysed during the current study.
