# Supplementary material for: Isolation of the new polyketide (−)-R-talaropinophiloic acid guided by an integrated metabolomics-dereplication approach
Source: World J Microbiol Biotechnol. 2026 Jul 23;42(8):427. doi: 10.1007/s11274-026-05150-2 (PMC13395978; doi:10.1007/s11274-026-05150-2)
Supplement: Supplementary file 1 — Supplementary Material 1 [file 11274_2026_5150_MOESM1_ESM.docx]

Supplementary Material

**Isolation of the New Polyketide (−)-*R*-Talaropinophiloic Acid Guided by an Integrated Metabolomics-Dereplication Approach**

Marcus Vinicius Almeida Marques,^a^ Andresa Hiromi Sakai,^b^ Lucas Haidar Martorano,^c^ Viviani N. Takahashi,^d^ Juliana Mara Serpeloni,^b^ Fernando Martins dos Santos Júnior,^c^ Jorge M. David,^a^ and Eliane O. Silva^a,*^

*^a^ Department of Organic Chemistry, Institute of Chemistry, Federal University of Bahia, Salvador 40170-115, Bahia, Brazil*

*^b^ Department of General Biology, Center for Biological Sciences, State University of Londrina, Londrina 86057-970, Paraná, Brazil*

*^c^ Department of Organic Chemistry, Institute of Chemistry, Federal Fluminense University, Niterói 24020-141, Rio de Janeiro, Brazil*

*^d^ Department of Chemistry, Faculty of Philosophy, Sciences and Letters of Ribeirão Preto, University of São Paulo, Ribeirão Preto 14040900, São Paulo, Brazil*

*Corresponding author:

elianeos@ufba.br (E.O. Silva)

Department of Organic Chemistry, Institute of Chemistry, Federal University of Bahia (UFBA), Barão de Jeremoabo 147, 40170-115 Salvador, Bahia, Brazil

A

B

C

D

E

F

**Fig. S1** Base peak chromatogram (100–1000 Da) from the extract of J1, J5, J6, J7, J9, and J13 strains (A-F, respectively) growth in PDA medium. Data acquired at electrospray ionization in positive mode.

| (a) | (b) |
| --- | --- |
| 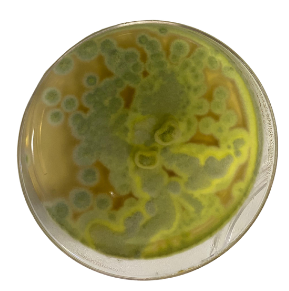 | **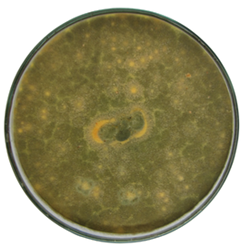** |

**Fig. S2** Macroscopic morphology of 7-day cultures of Talaromyces pinophilus J6 grown on potato dextrose agar with (a) and without (b) ammonium sulfate


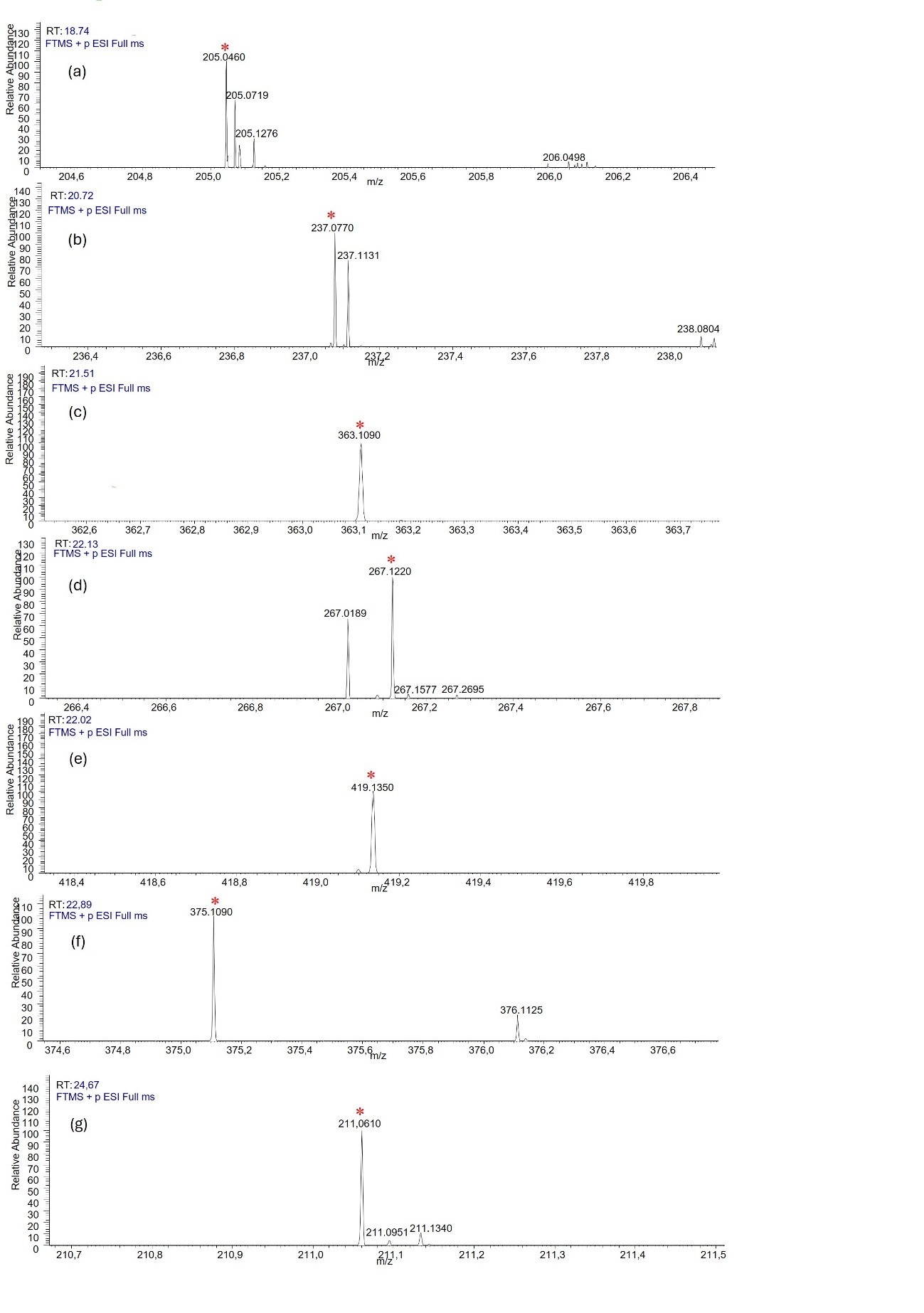


**Fig. S3** MS1 spectra acquired in positive ion mode of 3,4-dihydroxyphenylacetic acid methyl ester (**a**), 3-(hydroxymethyl)-6,8-dimethoxy-2*H*-chromen-2-one (**b**), talaromycolide A (**c**), actofunicone (**d**), amestolkin A (**e**), funicone (**f**), and rubralide C (**g**). The ions corresponding to each annotated compound are indicated in the spectra by red asterisks.

**Table S1.** Complete list of features exclusively detected in *Talaromyces pinophilus* J6 cultivated on PDA_AS medium. The seven annotated natural products (**a**–**g**), selected based on their low mass error, the presence of common positive-ion adducts under the employed analytical conditions, and an MZmine match score greater than 0.5 against our in-house library, are highlighted in gray. Their annotation was initially supported by MS1 data and subsequently strengthened through MS2 spectral analysis and the proposal of fragmentation pathways. The compounds isolated in this study are highlighted in green. ‘Unknown’ indicates that the feature was not detected, whereas ‘Detected’ indicates that the feature was observed.

| **row identity**  data bank ID:adduct:score | **m/z** | **retention time** | **J6_PDA** Feature status | **J6_PDA_ST** Feature status | **J6_PDA_AS** Feature status | **J6_PDA_AS** Peak area |
| --- | --- | --- | --- | --- | --- | --- |
| 864: [M+NH4]+: 0.617 | 136.06235 | 6.2624 | UNKNOWN | UNKNOWN | DETECTED | 2137533.5 |
| 711: [M+NH4]+: 0.812 | 215.10357 | 12.398 | UNKNOWN | UNKNOWN | DETECTED | 2579876.5 |
| 697: [M+NH4]+: 0.861 | 126.05565 | 12.702 | UNKNOWN | UNKNOWN | DETECTED | 5250707.5 |
| 646: [M+H]+: 0.867 | 152.07127 | 12.702 | UNKNOWN | UNKNOWN | DETECTED | 3569922.5 |
| 645: [M+H]+: 0.697 | 190.04835 | 12.702 | UNKNOWN | UNKNOWN | DETECTED | 7203956.5 |
| 820: [M+H]+: 0.445 | 239.06749 | 14.508 | UNKNOWN | UNKNOWN | DETECTED | 3510277.5 |
| 120: [M+NH4]+: 0.762 | 216.12422 | 15.554 | UNKNOWN | UNKNOWN | DETECTED | 3348312.8 |
| 705: [M+NH4]+: 0.787 | 190.10849 | 15.639 | UNKNOWN | UNKNOWN | DETECTED | 4433511.5 |
| 880: [M+NH4]+: 0.761 | 224.09293 | 15.767 | UNKNOWN | UNKNOWN | DETECTED | 875192.2 |
| 756: [M+H]+: 0.813 | 157.05051 | 16.018 | UNKNOWN | UNKNOWN | DETECTED | 4531233.5 |
| 208: [M+H]+: 0.706 | 169.04807 | 16.560 | UNKNOWN | UNKNOWN | DETECTED | 8055467 |
| 166: [M+NH4]+: 0.668 | 318.09888 | 16.812 | UNKNOWN | UNKNOWN | DETECTED | 1825431 |
| 576: [M+NH4]+: 0.746 | 241.15593 | 17.149 | UNKNOWN | UNKNOWN | DETECTED | 1013323.3 |
| 849: [M+H]+: 0.762 | 193.05077 | 18.134 | UNKNOWN | UNKNOWN | DETECTED | 6705788.5 |
| 847: [M+H]+: 0.770 | 207.02763 | 18.134 | UNKNOWN | UNKNOWN | DETECTED | 5029270 |
| 669: [M+K]+: 0.447 | 265.11728 | 18.497 | UNKNOWN | UNKNOWN | DETECTED | 4950071 |
| 109: [M+H]+: 0.809 | 181.05053 | 18.537 | UNKNOWN | UNKNOWN | DETECTED | 18381566 |
| 278: [M+NH4]+: 0.729 | 388.14107 | 18.697 | UNKNOWN | UNKNOWN | DETECTED | 1210966.5 |
| **a** 675: [M+Na]+: 0.776 | 205.0460 | 18.737 | UNKNOWN | UNKNOWN | DETECTED | 1801531.1 |
| 874: [M+NH4]+: 0.750 | 204.12428 | 18.777 | UNKNOWN | UNKNOWN | DETECTED | 6991657.5 |
| 749: [M+H]+: 0.738 | 246.0774 | 18.897 | UNKNOWN | UNKNOWN | DETECTED | 34431060 |
| 723: [M+H]+: 0.782 | 239.09029 | 18.937 | UNKNOWN | UNKNOWN | DETECTED | 3620280.2 |
| 750: [M+H]+: 0.755 | 202.08503 | 18.976 | UNKNOWN | UNKNOWN | DETECTED | 2186877.8 |
| 117: [M+H]+: 0.806 | 211.06107 | 18.976 | UNKNOWN | UNKNOWN | DETECTED | 6133644 |
| 428: [M+H]+: 0.669 | 321.09853 | 18.976 | UNKNOWN | UNKNOWN | DETECTED | 3847950.5 |
| 714: [M+H]+: 0.878 | 343.08062 | 18.976 | UNKNOWN | UNKNOWN | DETECTED | 1507671.5 |
| 798: [M+NH4]+: 0.676 | 302.10394 | 19.055 | UNKNOWN | UNKNOWN | DETECTED | 5636771.5 |
| 763: [M+K]+: 0.939 | 277.11974 | 19.214 | UNKNOWN | UNKNOWN | DETECTED | 6906535 |
| 824: [M+NH4]+: 0.880 | 354.09661 | 19.214 | UNKNOWN | UNKNOWN | DETECTED | 8593076 |
| 722: [M+H]+: 0.757 | 211.09526 | 19.373 | UNKNOWN | UNKNOWN | DETECTED | 15109078 |
| 839: [M+Na]+: 0.668 | 329.06488 | 19.574 | UNKNOWN | UNKNOWN | DETECTED | 1238988.4 |
| 3: [M+K]+: 0.858 | 432.16765 | 19.967 | UNKNOWN | UNKNOWN | DETECTED | 5636684.5 |
| 826: [M+H]+: 0.865 | 341.0649 | 20.006 | UNKNOWN | UNKNOWN | DETECTED | 1491232.8 |
| 244: [M+NH4]+: 0.559 | 400.14133 | 20.006 | UNKNOWN | UNKNOWN | DETECTED | 3326324.5 |
| 827: [M+H]+: 0.909 | 369.09642 | 20.564 | UNKNOWN | UNKNOWN | DETECTED | 7441919.5 |
| 279: [M+NH4]+: 0.712 | 374.12535 | 20.564 | UNKNOWN | UNKNOWN | DETECTED | 10174723 |
| 529: [M+H]+: 0.753 | 195.10033 | 20.603 | UNKNOWN | UNKNOWN | DETECTED | 9675234 |
| 139: [M+H]+: 0.767 | 265.10824 | 20.603 | UNKNOWN | UNKNOWN | DETECTED | 2201312.5 |
| 91: [M+H]+: 0.819 | 251.0923 | 20.683 | UNKNOWN | UNKNOWN | DETECTED | 2133682.2 |
| 798: [M+NH4]+: 0.676 | 302.10394 | 19.055 | UNKNOWN | UNKNOWN | DETECTED | 5636771.5 |
| 302: [M+NH4]+: 0.778 | 266.13757 | 20.683 | UNKNOWN | UNKNOWN | DETECTED | 1390973.5 |
| **b** 90: [M+H]+: 0.756 | 237.07699 | 20.722 | UNKNOWN | UNKNOWN | DETECTED | 1985499.2 |
| 265: [M+NH4]+: 0.648 | 360.14593 | 20.762 | UNKNOWN | UNKNOWN | DETECTED | 6682047 |
| 156: [M+H]+: 0.620 | 389.16137 | 20.762 | UNKNOWN | UNKNOWN | DETECTED | 1221826 |
| 880: [M+H]+: 0.769 | 207.06634 | 20.842 | UNKNOWN | UNKNOWN | DETECTED | 2982268.2 |
| 185: [M+H]+: 0.918 | 429.15397 | 20.842 | UNKNOWN | UNKNOWN | DETECTED | 2888913 |
| 647: [M+H]+: 0.695 | 195.06671 | 20.961 | UNKNOWN | UNKNOWN | DETECTED | 1848509 |
| 592: [M+K]+: 0.863 | 391.15964 | 21.435 | UNKNOWN | UNKNOWN | DETECTED | 2148652.2 |
| 575: [M+H]+: 0.791 | 183.09993 | 21.474 | UNKNOWN | UNKNOWN | DETECTED | 774721.6 |
| 774: [M+NH4]+: 0.657 | 390.15644 | 21.474 | UNKNOWN | UNKNOWN | DETECTED | 9727725 |
| **c** 114: [M+H]+: 0.717 | 363.10889 | 21.513 | UNKNOWN | UNKNOWN | DETECTED | 6734015 |
| 549: [M+H]+: 0.869 | 385.09112 | 21.513 | UNKNOWN | UNKNOWN | DETECTED | 6783180 |
| 771: [M+NH4]+: 0.871 | 192.06617 | 21.709 | UNKNOWN | UNKNOWN | DETECTED | 5632537.5 |
| 870: [M+K]+: 0.883 | 390.14598 | 21.709 | UNKNOWN | UNKNOWN | DETECTED | 27118170 |
| 207: [M+H]+: 0.742 | 291.0876 | 21.749 | UNKNOWN | UNKNOWN | DETECTED | 1698877 |
| 297: [M+H]+: 0.711 | 317.06702 | 21.749 | UNKNOWN | UNKNOWN | DETECTED | 7723254.5 |
| 611: [M+NH4]+: 0.692 | 332.1144 | 21.866 | UNKNOWN | UNKNOWN | DETECTED | 4373645 |
| 154: [M+H]+: 0.701 | 403.14027 | 21.866 | UNKNOWN | UNKNOWN | DETECTED | 3469163 |
| 198: [M+H]+: 0.845 | 155.10745 | 21.943 | UNKNOWN | UNKNOWN | DETECTED | 6951705.5 |
| **d** 136: [M+H]+: 0.637 | 419.13549 | 22.021 | UNKNOWN | UNKNOWN | DETECTED | 3001251.2 |
| **e** 368: [M+H]+: 0.785 | 267.1216 | 22.138 | UNKNOWN | UNKNOWN | DETECTED | 3553592.5 |
| 323: [M+Na]+: 0.155 | 405.15624 | 22.295 | UNKNOWN | UNKNOWN | DETECTED | 2666925 |
| 734: [M+H]+: 0.790 | 185.04553 | 22.373 | UNKNOWN | UNKNOWN | DETECTED | 1501829.2 |
| 351: [M+H]+: 0.762 | 249.07697 | 22.373 | UNKNOWN | UNKNOWN | DETECTED | 21168118 |
| 15: [M+K]+: 0.730 | 349.09351 | 22.373 | UNKNOWN | UNKNOWN | DETECTED | 34388988 |
| 214: [M+H]+: 0.737 | 223.09516 | 22.491 | UNKNOWN | UNKNOWN | DETECTED | 7368954 |
| 416: [M+Na]+: 0.609 | 443.1332 | 22.610 | UNKNOWN | UNKNOWN | DETECTED | 707472.1 |
| 387: [M+H]+: 0.783 | 283.11649 | 22.887 | UNKNOWN | UNKNOWN | DETECTED | 4211945 |
| **f** 133: [M+H]+: 0.666 | 375.10914 | 22.887 | UNKNOWN | UNKNOWN | DETECTED | 14816281 |
| 146: [M+]+: 0.753 | 397.09124 | 22.887 | UNKNOWN | UNKNOWN | DETECTED | 9575312 |
| 162: [M+H]+: 0.266 | 825.16985 | 23.043 | UNKNOWN | UNKNOWN | DETECTED | 26036162 |
| 111: [2M+H]+: 0.434 | 657.19951 | 23.082 | UNKNOWN | UNKNOWN | DETECTED | 1300383.2 |
| 903: [M+K]+: 0.403 | 404.14397 | 23.278 | UNKNOWN | UNKNOWN | DETECTED | 1516457.9 |
| 413: [M+H]+: 0.997 | 401.12306 | 23.437 | UNKNOWN | UNKNOWN | DETECTED | 1654892.6 |
| 253: [M+H]+: 0.926 | 413.15911 | 23.558 | UNKNOWN | UNKNOWN | DETECTED | 5950916.5 |
| 119: [M+H]+: 0.784 | 183.10266 | 23.761 | UNKNOWN | UNKNOWN | DETECTED | 2755833.8 |
| 418: [M+H]+: 0.782 | 417.11909 | 23.964 | UNKNOWN | UNKNOWN | DETECTED | 1339851.9 |
| 415: [M+Na]+: 0.782 | 427.10104 | 24.044 | UNKNOWN | UNKNOWN | DETECTED | 723205.5 |
| 209: [M+H]+: 0.863 | 109.06547 | 24.163 | UNKNOWN | UNKNOWN | DETECTED | 2451827.2 |
| 276: [M+H]+: 0.589 | 389.17277 | 24.163 | UNKNOWN | UNKNOWN | DETECTED | 3321278.2 |
| 164: [2M+NH4]+: 0.418 | 590.13222 | 24.403 | UNKNOWN | UNKNOWN | DETECTED | 2763903 |
| 375: [M+H]+: 0.404 | 239.0580 | 24.442 | UNKNOWN | UNKNOWN | DETECTED | 6511078 |
| **2** 411: [M+Na]+: 0.732 | 411.10538 | 24.442 | UNKNOWN | UNKNOWN | DETECTED | 836990140 |
| **1** 113: [M+Na]+: 0.726 | 379.07885 | 24.482 | UNKNOWN | UNKNOWN | DETECTED | 1064326.5 |
| 770: [M+H]+: 0.345 | 401.08998 | 24.521 | UNKNOWN | UNKNOWN | DETECTED | 8832310 |
| 219: [M+H]+: 0.805 | 197.04545 | 24.600 | UNKNOWN | UNKNOWN | DETECTED | 3958610.5 |
| 181: [M+Na]+: 0.963 | 375.0841 | 24.600 | UNKNOWN | UNKNOWN | DETECTED | 3517727.5 |
| **g** 135: [M+H]+: 0.091 | 211.0610 | 24.679 | UNKNOWN | UNKNOWN | DETECTED | 2687240.2 |
| 129: [M+Na]+: 0.315 | 187.07637 | 25.608 | UNKNOWN | UNKNOWN | DETECTED | 1672651.9 |
| 137: [M]+: 0.906 | 190.06347 | 25.608 | UNKNOWN | UNKNOWN | DETECTED | 2710075 |
| 772: [M+H]+: 0.772 | 233.08201 | 25.608 | UNKNOWN | UNKNOWN | DETECTED | 30356360 |
| 400: [M+Na]+: 0.558 | 407.11233 | 26.057 | UNKNOWN | UNKNOWN | DETECTED | 3658270.2 |
| 420: [M+Na]+: 0.606 | 409.12779 | 26.841 | UNKNOWN | UNKNOWN | DETECTED | 2167998.5 |
| 623: [M+Na]+: 0.643 | 413.26801 | 33.942 | UNKNOWN | UNKNOWN | DETECTED | 4542658.5 |
| 625: [M+H]+: 0.749 | 285.24367 | 34.835 | UNKNOWN | UNKNOWN | DETECTED | 1644876.1 |

**Fig. S4** 500 MHz ^1^H NMR spectrum of (-)-*R*-talaropinophiloic acid (**1**) registered in CD_3_OD


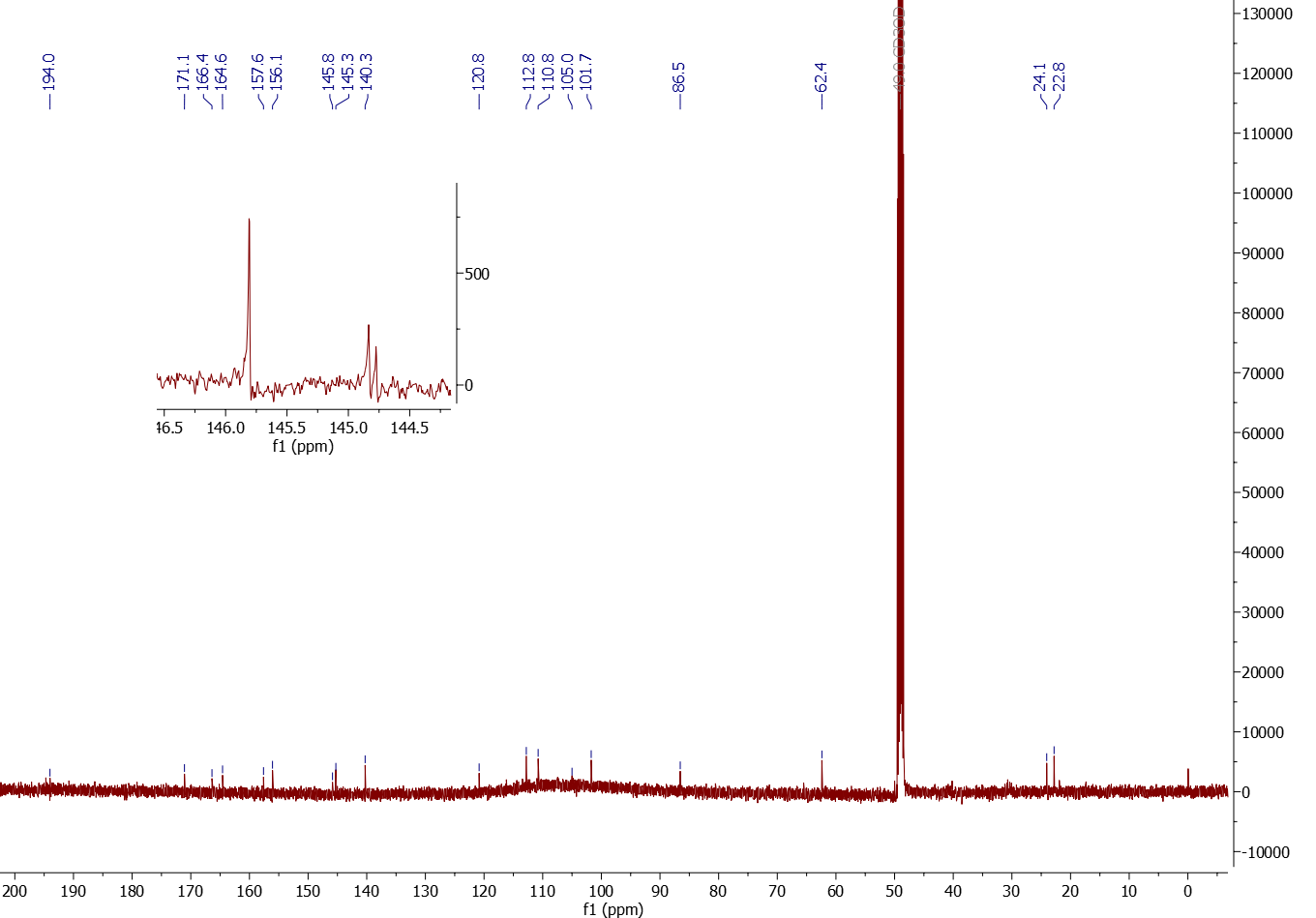

**Fig. S5** 125 MHz ^13^C spectrum of (-)-*R*-talaropinophiloic acid (**1**) registered in CD_3_OD

**Fig. S6** Heteronuclear HSQC (500 MHz for ^1^H; 125 MHz for ^13^C) contour map of (-)-*R*-talaropinophiloic acid (**1**) registered in CD_3_OD


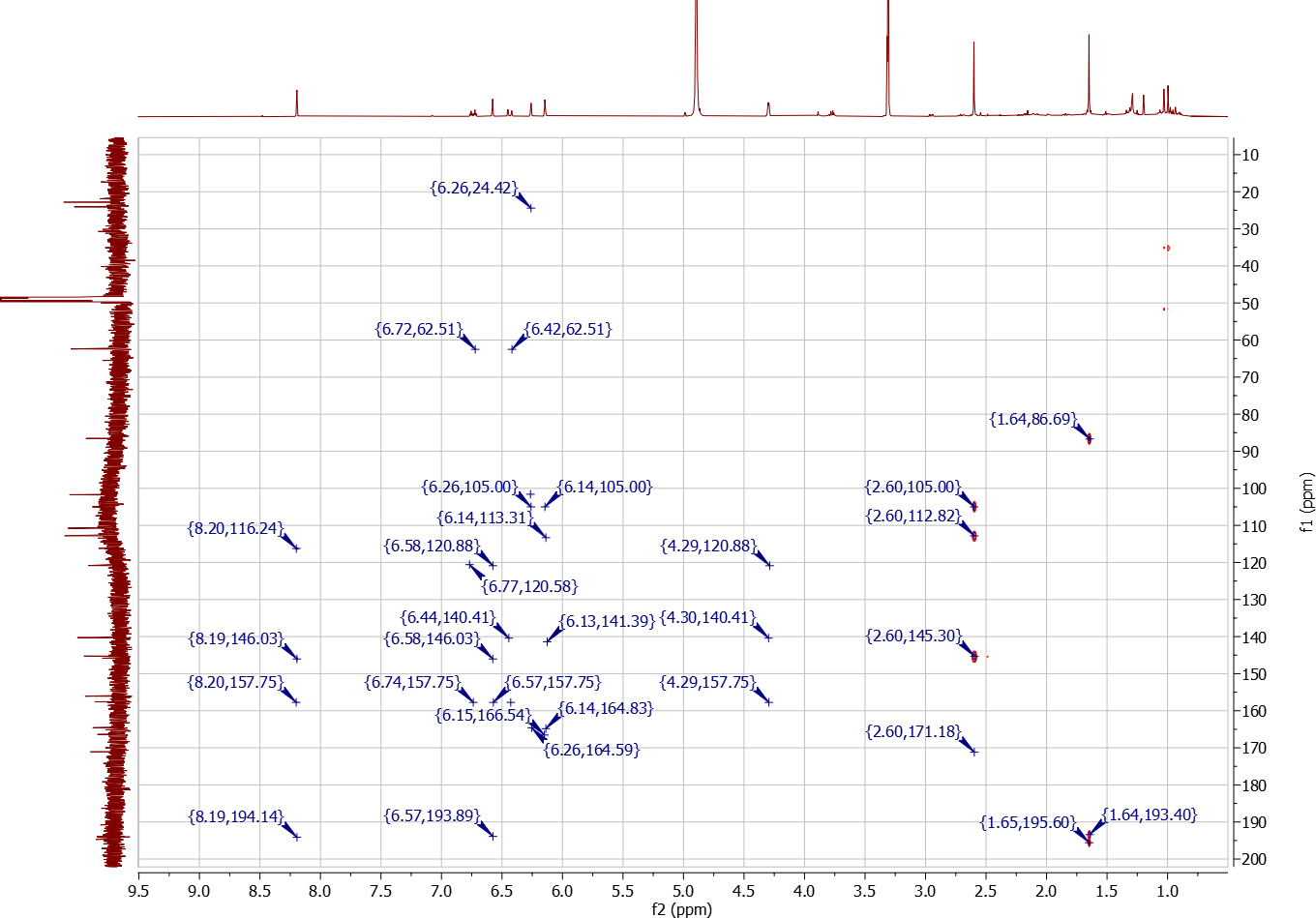

**Fig. S7** Heteronuclear HMBC (500 MHz for ^1^H; 125 MHz for ^13^C) contour map of (-)-*R*-talaropinophiloic acid (**1**) registered in CD_3_OD


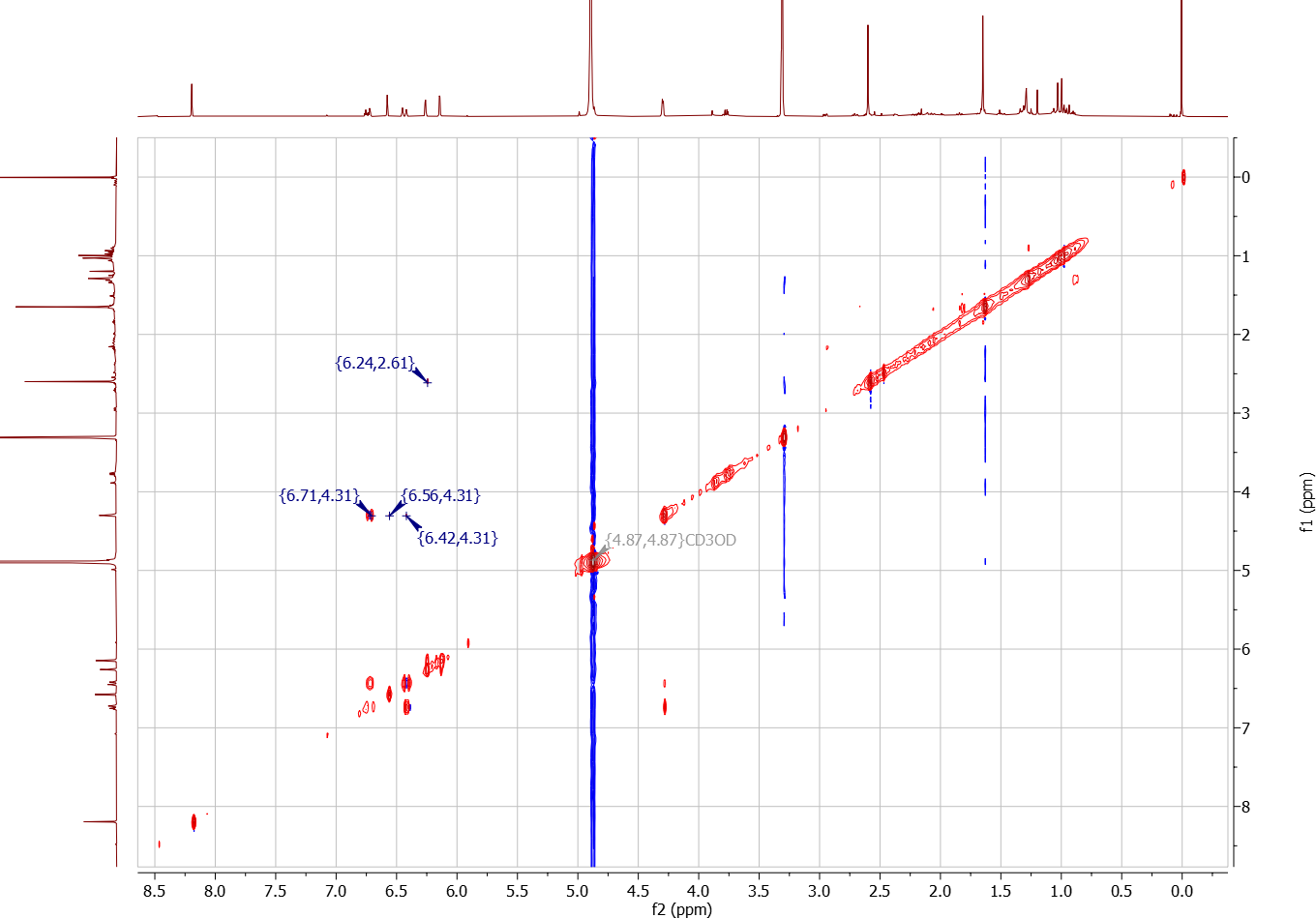

**Fig. S8** Homonuclear TOCSY (500 MHz) contour map of (-)-*R*-talaropinophiloic acid (**1**) registered in CD_3_OD

**Fig. S9** Homonuclear COSY (500 MHz) contour map of (-)-*R*-talaropinophiloic acid (**1**) registered in CD_3_OD

**Fig. S10** HRESIMS spectrum of (-)-*R*-talaropinophiloic acid (positive ion mode). HRMS *m*/*z* 357.0983 ([M+H]^+^; (C_19_H_16_O_7_)H^+^; calc. 357.0969; error 3.9 ppm).

**Fig. S11** 500 MHz ^1^H NMR spectrum of 3-*O*-methylfunicone (**2**) registered in CD_3_OD

**Fig. S12** 125 MHz ^13^C spectrum of 3-*O*-methylfunicone (**2**) registered in CD_3_OD

**Fig. S13** Heteronuclear HSQC (500 MHz for ^1^H; 125 MHz for ^13^C) contour map of 3-*O*-methylfunicone **(2**) registered in CD_3_OD

**Fig. S14** Heteronuclear HMBC (500 MHz for ^1^H; 125 MHz for ^13^C) contour map of 3-*O*-methylfunicone (**2**) registered in CD_3_OD

**Fig. S15** HRESIMS spectrum of 3-*O*-methylfunicone (positive ion mode). *m*/*z* 389.1244 ([M+H]^+^; (C_20_H_20_O_8_)H^+^; calc. 389.1231; error 3.3 ppm).
